# Determining selection free energetics from nucleotide pre-insertion to insertion in viral T7 RNA polymerase transcription fidelity control

**DOI:** 10.1093/nar/gkz213

**Published:** 2019-03-27

**Authors:** Chunhong Long, Chao E, Lin-Tai Da, Jin Yu

**Affiliations:** 1 Beijing Computational Science Research Center, Beijing 100193, China; 2 Shanghai Center for Systems Biomedicine, Shanghai JiaoTong University, Shanghai 200240, China

## Abstract

An elongation cycle of a transcribing RNA polymerase (RNAP) usually consists of multiple kinetics steps, so there exist multiple kinetic checkpoints where non-cognate nucleotides can be selected against. We conducted comprehensive free energy calculations on various nucleotide insertions for viral T7 RNAP employing all-atom molecular dynamics simulations. By comparing insertion free energy profiles between the non-cognate nucleotide species (rGTP and dATP) and a cognate one (rATP), we obtained selection free energetics from the nucleotide pre-insertion to the insertion checkpoints, and further inferred the selection energetics down to the catalytic stage. We find that the insertion of base mismatch rGTP proceeds mainly through an *off-path* along which both pre-insertion screening and insertion inhibition play significant roles. In comparison, the selection against dATP is found to go through an *off-path* pre-insertion screening along with an *on-path* insertion inhibition. Interestingly, we notice that two magnesium ions switch roles of leave and stay during the dATP *on-path* insertion. Finally, we infer that substantial selection energetic is still required to catalytically inhibit the mismatched rGTP to achieve an elongation error rate ∼10^−4^ or lower; while no catalytic selection seems to be further needed against dATP to obtain an error rate ∼10^−2^.

## INTRODUCTION

Transcription is the first step of gene expression in living organisms. It is directed by RNA polymerases (RNAPs) that transcribe genetic information from DNA to RNA, based on the Watson–Crick (WC) base pairing between the synthesizing RNA and the template DNA strand. The fidelity of transcription is highly crucial for maintaining normal gene expression, protein function, and cellular regulation. The transcription fidelity is controlled by nucleotide selections upon binding and incorporation along with proofreading during the RNAP transcription elongation (1–8). Without an RNAP, the template-based polymerization reaction proceeds extremely slowly and the error rate can hardly drop below ∼10^−2^, due to comparatively small free energy differences between cognate and non-cognate nucleotide additions (e.g. around 2–3 k_B_T). The acceleration of polymerization along with nucleotide selection and proofreading conducted via an RNAP can quench the transcription error rate down to ∼10^−4^–10^−7^ (9). In bacteriophage T7 RNAP transcription, the base mismatch error rate can be achieved at ∼10^−4^ or lower (10,11), without detection of the proofreading mechanism. Hence, the nucleotide selection in T7 RNAP plays a primary role in the viral transcription fidelity control, and it is of high interest to reveal how the nucleotide selection proceeds in structural and energetic detail.

**Figure 1.**
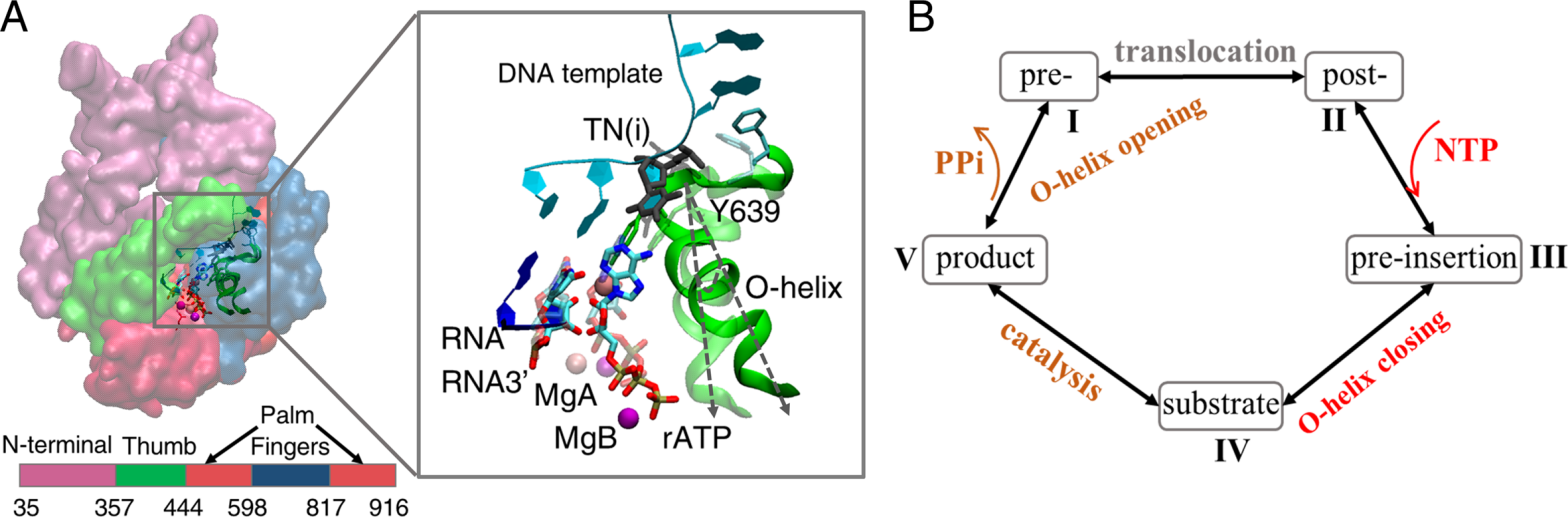
The structural and kinetic illustrations of the T7 RNAP elongation cycle. (**A**) Crystal structures of the T7 RNAP elongation complexes. In the left panel, the RNAP subdomains are colored red for the ‘palm’, green for the ‘thumb’, dark blue for the ‘fingers’ of the hand-like structure, and magenta for the N-terminal. The DNA template strand and RNA are shown in cyan and blue, respectively. The sequence regions of the respective subdomains are also given. In the right panel, an alignment between the pre-insertion structure (PDB 1S0V, non-transparent) (20) and the substrate insertion structure (PDB 1S76, transparent) (32) of T7 RNAP is provided, in a zoom-in view around the active site. An illustration of the O-helix rotation angle is provided, with the O-helix open in the pre-insertion state, and closed in the insertion state. (**B**) A kinetic scheme of the NAC of T7 RNAP. The NTP insertion process from the initial pre-insertion state (III) to the final substrate insertion state (IV) is our focus in this work.

The single-subunit T7 RNAP adopts a hand-like structure that appears common for viral RNAPs and a large class of DNA polymerases (DNAPs) (12–16) (see Figure 1A). In these polymerases, the fingers subdomain of the hand-like structure opens and closes throughout each nucleotide addition cycle (NAC) to allow insertion of an incoming nucleoside triphosphate (NTP), from an initial binding or pre-insertion site to the active site of RNAP. Presumably, an open structure of the single-subunit polymerase converts into a closed form upon a cognate NTP insertion, likely via a combined conformational selection and induced-fit mechanism of the nucleotide binding and insertion, e.g., as being suggested by studies of eukaryotic DNAP β (17). In comparison, non-cognate NTP binding or insertion may lead to an open or half-open intermediate state, as shown for DNAP I (18,19).

The nucleotide selection can happen at multiple checkpoints upon the nucleotide binding or insertion, prior to or during catalytic reaction (6). The experimental characterization on the stepwise selectivity of the RNAP had been conducted, e.g. for T7DNAP and a bacterial RNAP (2,16). In T7 RNAP, a pre-insertion complex had been obtained with a ‘semi-open’ conformation of the fingers subdomain (20). Since the WC base pairing was not observed in the crystal structure of the pre-insertion complex, it was not clear whether the nucleotide selection started from the initial binding or not. Nevertheless, our previous study indicated that the WC base pairing between the incoming NTP and the template transition nucleotide TN(i) (dTTP here) was able to form in an equilibrated solution structure from molecular dynamics (MD) simulation (21). Notably, a critical residue Tyr639 at the C-terminal end of an O-helix on the fingers subdomain interacted closely with the non-cognate nucleotide at pre-insertion. The close association with Tyr639 seemed to drag the non-cognate nucleotide to an ‘*off-path*’ pre-insertion configuration (22), in which the nucleotide could dissociate easily. Besides, the template TN(i) deviated significantly away from the non-cognate NTP in the ‘*off-path*’ configuration, while in a constructed ‘*on-path*’ pre-insertion configuration, TN(i) associated closely with the incoming NTP even if the WC base pairing was lack of (22). Thus, it seemed that essential nucleotide selection in T7 RNAP started early at the nucleotide pre-insertion stage, e.g., coordinated by this highly conserved ‘gating’ residue Tyr639 (23,24).

On the other hand, biochemical and kinetic studies have demonstrated that a slow step follows the initial NTP binding to allow for the nucleotide insertion (25). We have also shown generically that the slow or rate-limiting step can play a significant role in the fidelity control of the template-based polymerization (26). It is highly likely that the non-proofreading T7 RNAP relies on the slow nucleotide insertion to conduct substantial nucleotide selection, or transcription fidelity control. Hence, it is desirable to characterize the stepwise nucleotide selection in a structure-based and quantitative manner, particularly to the slow nucleotide insertion step, using free energy and related measures.

In current work, we employed intensive all-atom MD simulations above microseconds in aggregation to investigate the complete structural dynamics and free energetics of the nucleotide insertion, from a comparatively open-form pre-insertion structure, to a closed-form substrate or insertion structure of T7 RNAP that is ready for catalysis (see Figure 1B), for both the cognate and non-cognate nucleotides. By constructing the potential of mean forces (PMFs) using the umbrella sampling methodology (27–30), we not only provided free energy profiles for various nucleotides during the insertion process, but also derived correspondingly the nucleotide selection energetics from the pre-insertion to the insertion checkpoints (6). The PMFs were constructed along the collective coordinates of an essential set of atoms, which were regarded highly relevant to the nucleotide insertion. The essential set encompassed the majority of atoms involved in the open-to-close conformational transition of the RNAP fingers subdomain, the insertion NTP *per se*, and the corresponding template nucleotide. The collective coordinate was defined according to the difference between the root-mean-square deviations (*rmsds*) of a current structure from the respective pre-insertion and insertion reference structures. The non-cognate nucleotides included a mismatched ribo-nucleotide (rNTP) and a deoxy-ribonucleotide (dNTP). Following the previous clues (22), both an *on-path* and an *off-path* insertion processes of the non-cognate nucleotides were probed for the free energy calculations. Finally, we were able to infer the selection free energetics down to the catalytic stage by fitting with experimentally measured error rates via a chemical master equation (CME) approach onto the T7 RNAP elongation kinetics (26,31). This way, we completely characterized the fidelity control in this prototypical viral transcription system, with both classical structural dynamics and free energetic details.

Below, we present how we performed the free energy calculations using all-atom MD simulations: Mainly, we constructed individual PMFs for the cognate and non-cognate nucleotide insertion processes by performing the umbrella sampling simulations. Consequently, we obtained the activation free energies or barriers for those individual nucleotide insertions, along with representative conformations on the insertion paths. In particular, in order to align these individual PMFs together and determine the selection free energetics arising between the non-cognate and cognate nucleotides, we also calculated the relative binding free energies between them at the nucleotide pre-insertion, by performing alchemical MD simulations. In the end, we were able to demonstrate how nucleotide selection free energetics distributed from the pre-insertion to the insertion stage, by additionally incorporating previous knowledge on nucleotide dissociation at pre-insertion. Furthermore, we also address how we inferred the selection energetics for the catalytic stage in the elongation kinetic framework by numerically fitting the calculated elongation error rates with experimentally measured values.

## MATERIALS AND METHODS

Below we first show how we obtained initial and final structures for both the *on-path* and *off-path* nucleotide insertion simulations, along with general MD simulation setup. Then we illustrate the umbrella sampling method to construct the PMFs for individual nucleotide insertion processes. In our simulation system, the template DNA transition nucleotide (TN) at site *i* is a dTTP, so the cognate ribo-nucleotide is an rATP; a mismatched ribo-nucleotide rGTP and a deoxy-ribonucleotide dATP have been used as the non-cognate species in this study. Followed by that, we show how to determine the relative free energetics between the cognate and non-cognate nucleotides, by conducting alchemical simulations and obtaining the relative binding free energies between the rGTP/dATP *on-path* pre-insertion configuration and that of the cognate rATP. In the end, based on the nucleotide selection free energetics derived from the MD simulation results, we show how we calculated the elongation error rates according to the CME approach, and inferred the selection free energetics during catalysis.

### The initial and final structures of the insertion along with MD setup

The high-resolution structures of the T7 RNAP elongation complexes (protein, nucleic acids, or NAs, along with the NTP substrate) were captured by crystallization studies in two conformational states key to this study, the pre-insertion and the insertion complexes (with PDB codes 1S0V and 1S76, respectively) (20,32), as the initial and final reference structures of the nucleotide insertion, respectively. The missing residues were added based on the corresponding parts from other elongation complex structures in the post-translocation and product states (PDB:1MSW,1S77) (32,33). We also modified the original DNA/RNA sequences in the insertion state structure (1S76) to be consistent with that in the pre-insertion structure (1S0V).

Firstly, the initial pre-insertion structures of the cognate and non-cognate complexes, for both the *on-path* and *off-path* configurations were constructed (see Supplementary Figure S1A). The cognate rATP pre-insertion structure was obtained after an equilibrium MD simulation of 100 ns, starting from the crystal structure of the pre-insertion complex (PDB: 1S0V) (20). The crystal waters within 10 Å of the rATP molecule were retained. After the 100-ns equilibrium simulation of the rATP pre-insertion complex, ∼20 windows of 100-ns alchemical simulations each were conducted to transform rATP gradually into rGTP and dATP, respectively, so that the dATP and rGTP *on-path* initial structures were obtained in the last window, along with the relative binding free energies between the *on-path* dATP/rGTP and rATP at pre-insertion (addressed later), as conducted in our previous alchemical simulation study (22). In comparison, to obtain the *off-path* initial structures, the cognate rATP in the pre-insertion crystal complex was directly converted into the non-cognate rGTP and dATP, respectively; after energy minimization, 100-ns equilibrium simulations were conducted for the respective non-cognate pre-insertion complexes to obtain the *off-path* initial structures, as conducted in another of our previous simulation studies (21).

Then for the final substrate insertion structures, the cognate rATP structure was obtained from the equilibrated complex of the crystal structure (PDB: 1S76) (32); the non-cognate rGTP and dATP substrate structures were constructed by directly converting rATP into rGTP and dATP, respectively, based on the insertion crystal structure, and were then subjected to MD equilibration (see Supplementary Figure S1B).

All MD simulations were performed using GROMACS-5.1.2 package (34,35) and the Amber99sb force field with ParmBsc0 nucleic acid parameters was used (36–39). The rATP/rGTP/dATP parameters were obtained from Carlson *et al.* (40). For the equilibrium MD simulation, the RNAP complex was solvated with explicit TIP3P water (41) in a cubic box with a size of ∼120 Å, and the minimum distance from the protein to the wall was set to 13 Å. A larger simulation box (up to ∼165 Å) with expanded number of water molecules was tested, with no further energetic changes of protein-DNA interactions within the RNAP pre-insertion complex found, and no further essential conformation changes such as the O-helix rotational motions either. To neutralize the system and make the salt concentration 0.1 M with counter ions, 176 Na^+^ ions and 142 Cl^−^ ions were added. Two magnesium ions were kept as that from respective crystal structures of the pre-insertion and insertion complexes (20,32). The full simulation system contained ∼156 000 atoms, with ∼140 000 atoms for the water molecules. For all simulations, the cut-off of van der Waals (vdW) and the short-range electrostatic interactions were set to 9 and 10 Å, respectively. Particle-mesh-Ewald (PME) method (42,43) was used to evaluate the long-range electrostatic interactions. All MD simulations were run at 1 bar and 310 K using the Parrinello−Rahman Barostat (44,45) and the velocity rescaling thermostat (46), respectively. Before each of the equilibrium NPT simulation, we minimized the initial structure for 50 000 steps with the steepest-descent algorithm followed by 100-ps NVT MD simulation. The time step was 2 fs and the neighbor list was updated every 5 steps.

### Umbrella sampling simulations

In order to obtain the free energy profiles or PMFs between the pre-insertion and the insertion states of T7 RNAP, for both the cognate and non-cognate substrate species, we launched reaction paths along the changes of the *rmsd* of an essential set of atoms (see ${\rm{\delta }}rmsd$ and the atom selection addressed later). The choice of this reaction coordinate is due to such $\ {\rm{\delta }}rmsd$ being highly collective and relevant to the substantial conformational changes involved in the nucleotide insertion. To avoid too large deviations from the two-end structures at the pre-insertion and insertion states, both forward (pre-insertion to insertion) and backward (insertion to pre-insertion) paths were launched, and the first half of these two paths were merged into one insertion path. Subsequently, we generated a series of configurations along the insertion paths for the cognate rATP and non-cognate rGTP/dATP (*on-path* and *off-path*); then each of these configurations was subjected to the umbrella sampling simulation with force constraints (47). Finally, the PMFs along the ${\rm{\delta }}rmsd$ reaction coordinate were obtained by using a weighted histogram analysis method (WHAM) (28,30). The technical details are provided below.

#### Launch the initial nucleotide insertion pathway along the reaction coordinate

Based on the modeled pre-insertion and insertion structures, we obtained the initial forward and backward insertion paths by using a modified version of the Climber algorithm (48). In the respective paths, the insertion and the pre-insertion structures were set as the final reference structures. We selected Cα atoms of five helices in the fingers subdomain (residue 627–639, 568–589, 612–624, 642–660 and 669–687) and heavy atoms of substrate rATP/rGTP/dATP and template TN(*i*) as the morphed regions. These regions undergo substantial conformational changes as observed from the crystal structures (see Supplementary Figure S2A). We excluded the flexible loop regions on the fingers subdomain that may involve irrelevant motions. Indeed, we chose the above morphed region to serve as a minimum set to be essential for the nucleotide insertion. Inclusion of a larger set of atoms, e.g. from the DNA or RNA strand, the final morphed structures actually demonstrated larger deviations from the insertion target (see Supplementary Figure S2B and C), likely due to extra fluctuations brought about by the DNA/RNA strand. In the Climber simulation, external forces were then applied to the atoms in the morphed region, whereas the remainder of the system responded to the structural changes in the morphed region; the target number of morphing cycles was set to 700, and each morphing cycle consisted of 100 iterations of morphing, with 10 steps of conjugate gradient energy minimization for each 10 iterations; the minimum distance (or the *rmsd*) to the target structure was set to 0.4 Å and was reached after 400 morphing cycles.

In launching the reaction paths for the subsequent umbrella sampling simulations, we used $\delta rmsd$ as the reaction coordinate, which is defined as $\delta rmsd = rmsd( {X,\,{X_{init}}} ) - rmsd( {X,\,{X_{final}}} )$, where *X* represents a collective coordinate of our selected structural regions (specified above or see Supplementary Figure S2A); *X_init_* and *X_final_* refer to corresponding coordinates of the reference structures near the equilibrium initial pre-insertion and final insertion complexes, respectively; and *rmsd* is measured between the two set of coordinates denoted inside the parenthesis. ${\rm{\ }}\delta rmsd$ has been successfully used as an order parameter or reaction coordinate to characterize the transition pathway between a pair of structures in biomolecular simulations (49,50). Along the $\delta rmsd{\rm{\ }}$reaction coordinate here, the interval distance between two neighboring windows was set to 0.1 Å, so that 27 windows were obtained for the cognate rATP (or non-cognate rGTP/dATP *on-path*) insertion as $\delta rmsd{\rm{\ }}$ranges from –1.3 to 1.3 Å, and 45 or 53 windows were obtained for the non-cognate rGTP/dATP *off-path* insertion as $\delta rmsd$ spans from –2.2 to 2.2 Å or –2.6 to 2.6 Å. The choice of the number of windows ensured sufficient overlaps between neighboring simulation windows that is required for the construction of the PMF (see below).

#### Conducting the umbrella sampling simulations

The umbrella sampling simulations were performed by using PLUMED (51) to add force constraints on the collective $\delta rmsd$ coordinate, with each window simulated for ∼40 ns. The same MD setup was used as specified above.

The 27 structures (45 or 53 structures for the non-cognate *off-path*) along the reaction path were subjected to the umbrella sampling simulations with the forces applied to the selected structure regions according to $F = k( {\delta rmsd - \delta rmsd0} )$, where $\delta rmsd0$ was the specified value for the simulation window, around which $\delta rmsd$ was restrained, and *k* adopted a value at 210 000 ${\rm kJ}/({\rm mol} \cdot {\rm n}{{\rm m}^2})$ for regular windows (or 10 times larger for windows near the free energy barrier, or 10 times smaller for windows near the free energy minima).

#### Constructing the PMFs and error analyses

The PMFs or the free energy profiles along the $\delta rmsd$ reaction coordinate were obtained by using the WHAM (28,30) on a series of 40-ns trajectories from the umbrella simulation windows, while the first 10-ns pre-equilibration data were not used. The WHAM was used to transform the biased sampling results to the unbiased sampling ones. Basically, one calculates the unbiased probabilities from the biased samplings by using the equation below

(1) \begin{equation*}{P_i}\left( {\delta rmsd} \right) \propto {e^{\frac{{ - \frac{1}{2}k{{\left( {\delta rmsd - \delta rmsd0} \right)}^2}}}{{{k_B}T}}}}P_i^{\prime}\left( {\delta rmsd} \right)\end{equation*}

where ${P_i}( {\delta rmsd} )$ and $P_i^{\prime}( {\delta rmsd} )$ are the unbiased and biased probabilities, respectively. $\frac{1}{2}k(\delta rmsd - \delta rmsd{ 0 )^2}$ is the harmonic restraint potential. Note that the full probability distribution *P*$(\delta rmsd)$ is not simply the addition of the individual probability distributions obtained from each window, but a linear combination of them. Lastly, according to $G( {\delta rmsd} ) = - {k_B}TlnP( {\delta rmsd} )$, the free energy profile *G* along the coordinate$\ \delta rmsd$ or the PMF was obtained.

During the construction of PMF by using WHAM, we performed bootstrapping in order to estimate errors (52). The WHAM computes the PMF based on all the $\delta rms{d_i}$(t) obtained from the simulation windows (*i* = 1, 2,…, *n*). In order to get the bootstrapped trajectories $\ \delta rms{d_{b,i}}( t )$, one re-samples the $\delta rms{d_i}$ in each window. Each bootstrapped trajectory $\delta rms{d_{b,i}}( t )$ produces a new histogram ${h_{b,i}}( {\delta rmsd} )$. Then, via the WHAM procedure, one computes a bootstrapped PMF ${G_b}( {\delta rmsd} )$ based on the new set of *n* histograms ${h_{b,i}}$. The whole process is repeated *N* times (*N* = 200 used), generating *N* bootstrapped PMFs ${G_{b,j}}( {\delta rmsd} )$ (*j* = 1, 2,…, *N*). The uncertainty of the PMF is then estimated by the standard deviation calculated by the *N* bootstrapped PMFs (52).

(2) \begin{equation*}\begin{array}{@{}*{1}{l}@{}} {{\sigma _{PMF}}(\delta rmsd)}\\ { = {{\left[ {{{(N - 1)}^{ - 1}}{{\sum\nolimits_{j = 1}^N {\left( {{G_{b,j}}\left( {\delta rmsd} \right) - \left\langle {{G_b}(\delta rmsd)} \right\rangle } \right)} }^2}} \right]}^{\frac{1}{2}}}} \end{array}\end{equation*}

### Evaluating the relative binding free energies between the *on-path* non-cognate and cognate NTPs at pre-insertion

Though we obtained the *on-path* and *off-path* PMFs for the non-cognate rGTP/dATP insertion individually, how these PMFs deviated from that of the cognate rATP at the beginning of the insertion process needed to be determined. Accordingly, we calculated the relative binding free energies between the *on-path* non-cognate substrate species and the cognate one at the pre-insertion site, by using the alchemical method illustrated below.

#### The relative binding free energy via a thermodynamic cycle

For the substrates or ligands rGTP/dATP (non-cognate or *nc*) and rATP (cognate or *c*) at the pre-insertion site, the relative binding free energies between them can be obtained via a thermodynamic cycle as

(3) \begin{equation*}\Delta \Delta {G_b} \equiv \Delta {G_b}^{nc} - \Delta {G_b}^c = \Delta G_a^{pro} - \Delta G_a^{sol}\end{equation*}

where $\Delta {G_b}^{nc}$ and $\Delta {G_b}^c$ are the binding free energies of the non-cognate and cognate nucleotides at the pre-insertion site, respectively; $\Delta G_a^{pro}$ and $\Delta G_a^{sol}$ are the free energies evaluated by transforming the cognate rATP alchemically into the non-cognate rGTP/dATP in the protein complex and in the free solution, respectively (22). Then the free energy perturbation (FEP) method (53) was used to calculate the alchemical energy. In order to accurately evaluate the free energy change, the bidirectional sampling using the Bennett acceptance ratio (BAR) method (54) was implemented in the GROMACS package (34,35,55), with both forward and backward alchemical transformations performed in the simulation (56).

#### The implementation of the alchemical simulations

In the alchemical simulation, three dummy atoms were added to rATP in order to convert rATP (rGTP/dATP) to rGTP/dATP (rATP) in the forward (backward) direction. For the forward simulation, the transformation of the cognate into the non-cognate nucleotide was controlled via a parameter λ from 0 to 1, with an increment of 0.05, and it was *vice versa* for the backward path (22).

During the simulation, the vdW and electrostatic interaction were simultaneously changed. The LINCS algorithm was used to constrain all the chemical bonds (57). In the free-solution simulation, NTP was solvated in a cubic box with ∼4000 TIP3P water molecules, the minimum distance from NTP to the wall was set to 10 Å. Ten Na^+^ ions and eight Cl^−^ ions were added to keep the ionic concentration at 0.1 M and neutralize the system. The simulations with the protein complex were the same as specified above. In each direction, 21 windows of 100-ns simulation each were carried out in the protein complex and in the free solution, respectively (22).

### Deriving the elongation error rate from the stepwise selection energetics

Here, we use a five-state kinetic scheme including the pre-translocation state (I), post-translocation state (II), pre-insertion state (III), substrate insertion state (IV), and product state (V) to describe an RNAP elongation or NAC cycle (see Figure 1B and the linear scheme with rates denoted below). Importantly, the RNAP can differentiate the cognate and non-cognate NTP species upon the nucleotide binding as well as during the insertion and catalysis processes (6,25,26).

(4) \begin{equation*}{\rm{I}}\mathop {\underset{{{{\rm{k}}_{{\rm{II}} - }}}}{\overset{{{{\rm{k}}_{{\rm{I}} + }}}}{\rightleftharpoons}}}\limits^{{\rm{Translocation}}} \mathop {{\rm{II}}\underset{{{{\rm{k}}_{{\rm{III}} - }}}}{\overset{{{{\rm{k}}_{{\rm{II}} + }}}}{\rightleftharpoons}}}\limits^{{\rm{NTP}}\,{\rm{binding}}} \mathop {{\rm{III}}\underset{{{{\rm{k}}_{{\rm{IV}} - }}}}{\overset{{{{\rm{k}}_{{\rm{III}} + }}}}{\rightleftharpoons}}{\rm{IV}}}\limits^{{\rm{Insertion}}} \mathop {\underset{{{{\rm{k}}_{{\rm{V}} - }}}}{\overset{{{{\rm{k}}_{{\rm{IV}} + }}}}{\rightleftharpoons}}{\rm{V}}}\limits^{{\rm{Catalysis}}} \mathop {\underset{{{{\rm{k}}_{{\rm{I}} - }}}}{\overset{{{{\rm{k}}_{{\rm{V}} + }}}}{\rightleftharpoons}}{\rm{I^\prime}} }\limits^{{\rm{PPi}}\,{\rm{release}}} \end{equation*}

Correspondingly, there are four kinetic checkpoints upon the NTP binding (pre-insertion) and incorporation steps, as described in one our previous modeling work (6). The first selection checkpoint (*III→ II*) rejects non-cognate NTP immediately upon the NTP binding or pre-insertion, with a selection strength $\eta _{III}^ - \equiv \frac{{k_{III - }^{nc}}}{{k_{III - }^c}} = {e^{\Delta _b^ - /{k_B}T}}$, where $\Delta _b^ -$ is defined as the selection free energy at the pre-insertion state, or the difference between the dissociation free energy barriers of the cognate $( {\Delta E_d^c} )$ and the non-cognate $( {\Delta E_d^{nc}} )$ NTP $( {\Delta _b^ - \equiv \Delta E_d^c - \Delta E_d^{nc}} )$. The next selection checkpoint (*III → IV*) inhibits the non-cognate nucleotides from inserting into the active site, with a selection strength ${\rm{\ }}\eta _{III}^ + \equiv \frac{{k_{III + }^c}}{{k_{III + }^{nc}}} = {e^{\Delta _{in}^ + /{k_B}T}}$, where $\Delta _{in}^ +$ is defined as the insertion selection free energy, or the difference between the insertion free energy barriers of the non-cognate and the cognate NTP $( {\Delta _{in}^ + \equiv \Delta E_{in}^{nc} - \Delta E_{in}^c} )$. The third selection checkpoint (IV→ III) destabilizes the non-cognate nucleotides after being inserted at the substrate state *IV*$( {\eta _{IV}^ - \equiv \frac{{k_{IV - }^{nc}}}{{k_{IV - }^c}} = {e^{\Delta _{in}^ - /{k_B}T}}} )$, where $\Delta _{in}^ -$ is the difference between the free energy barriers to reverse the insertion process of the cognate and the non-cognate nucleotides $( {\Delta _{in}^ - \equiv \Delta E_{rev}^c - \Delta E_{rev}^{nc}} )$. The last selection checkpoint (IV → V) inhibits the catalytic reaction of the non-cognate nucleotides comparing to the cognate one $( {\eta _{IV}^ + \equiv \frac{{k_{IV + }^c}}{{k_{IV + }^{nc}}} = {e^{\Delta _c^ + /{k_B}T}}} )$, where $\Delta _c^ +$ is the catalytic selection free energy or the free energy barrier difference between the non-cognate and cognate nucleotide species during the catalysis $( {\Delta _c^ + \equiv \Delta E_{cat}^{nc} - \Delta E_{cat}^c} )$. In general, every checkpoint can play some role during the nucleotide selection $(\Delta >0)$, while an important checkpoint may contribute significantly $(\Delta \gg 0)$. Correspondingly, the populations and probability fluxes of the non-cognate and cognate species can be treated separately, with the respective fluxes or elongation rates as, e.g. ${J^{nc}} = P_V^{nc}{k_{V + }} - Err{P_I}{k_{I - }}$ and ${J^c} = P_V^c{k_{V + }} - ( {1 - Err} ){P_I}{k_{I - }}$, where $( {{P_I},{P_{II}},P_{III}^c,P_{III}^{nc},P_{IV}^c,P_{IV}^{nc},P_V^c,P_V^{nc}} )$ is the vector for the state population distributions, and *Err* denotes the elongation error rate. The error rate is defined as the ratio between the non-cognate and total elongation rates or fluxes as $Err \equiv {J^{nc}}/J$, with $J = {J^c} + {J^{nc}}$ being the total flux or elongation rate at the steady-state condition. More calculation details can be found in references (6,26). The mainly elongation kinetic parameters of T7 RNAP had been determined from previous biochemical and single molecule experiments (25,58). Since all the pre-catalytic selection energetics have been obtained from our current MD simulations and related studies, one can then calculate the elongation error rate using the above kinetic parameters and selection energetics, or one can derive the catalytic selection energy by fitting with error rates measured experimentally (10,11).

## RESULTS

We first present the PMF of the cognate rATP nucleotide insertion, constructed along the collective reaction coordinate $\delta rmsd$. The representative structures and a simulation movie along the rATP insertion path are provided. Then we also present the PMFs for the non-cognate nucleotides rGTP and dATP, along with the key structures and movies, following either the *on-path* or *off-path* insertion process. Furthermore, we show that by calculating the relative binding free energies between the non-cognate and cognate nucleotides at pre-insertion, we are able to align the above PMF profiles together, thus revealing the stepwise nucleotide selection energetics and corresponding structural dynamics from the pre-insertion to the insertion.

### Cognate rATP insertion: The O-helix resists closing during the nucleotide insertion but closes well upon the cognate rATP insertion

First, we examined the insertion energetics and structural dynamics of the cognate rATP by starting from the rATP pre-insertion structure. The WC base pairing between rATP and template TN(i) was not captured in the crystal structure of the pre-insertion complex (20). However, after ∼50 ns equilibrium MD simulation of the rATP pre-insertion structure, the WC base pairing formed, as reported from our previous study (21). Throughout the equilibrium simulation, Tyr639 from the C-terminus of the O-helix occupied around the active site, and the O-helix fluctuated around a comparatively open conformation (∼15 ± 2°).

**Figure 2.**
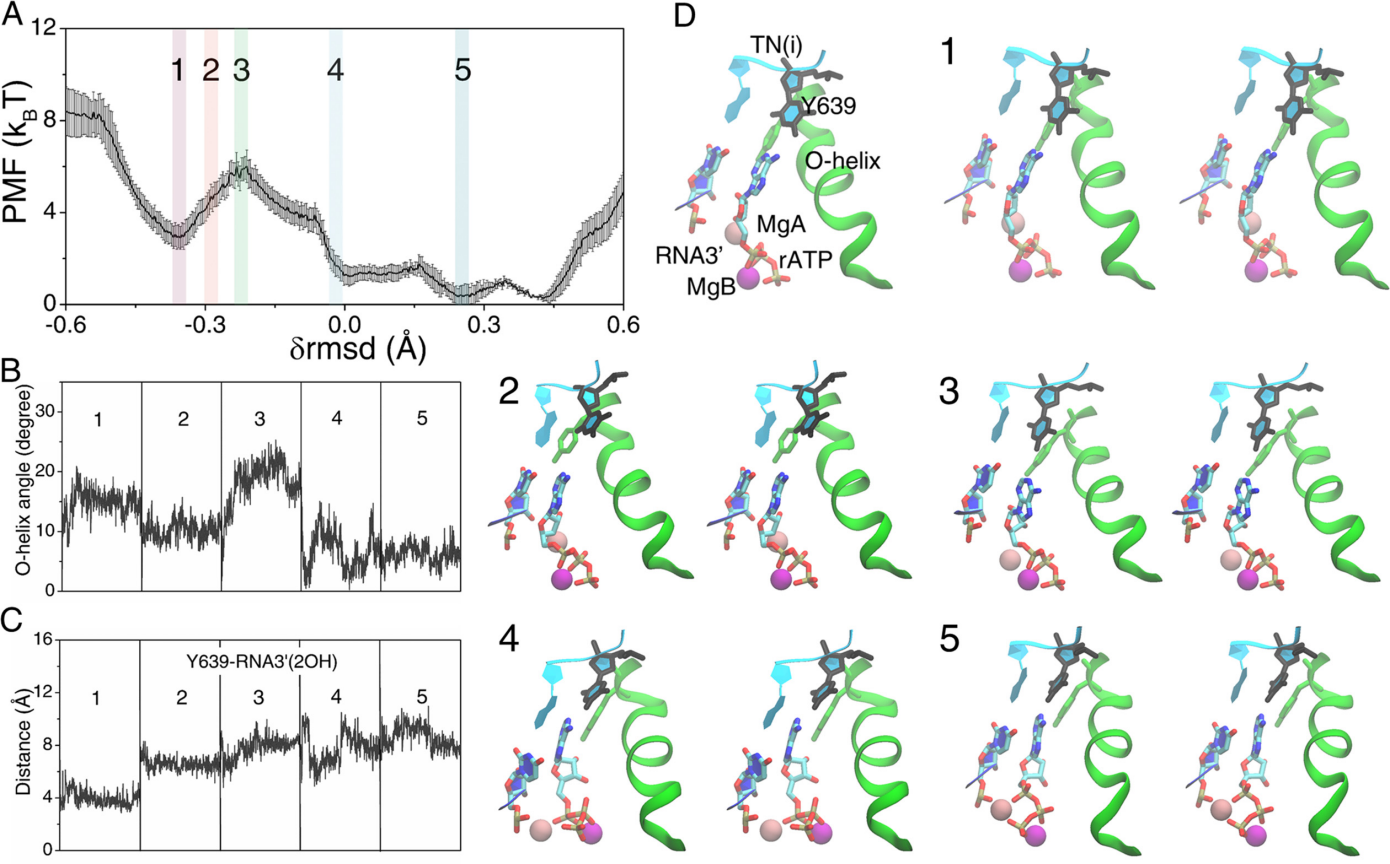
The PMF from the initial pre-insertion to the final substrate insertion state for the cognate rATP bound RNAP elongation complex and key structural measurements. (**A**) The PMF was constructed from the umbrella sampling simulations (29) along the collective reaction coordinate ${\rm{\delta rmsd}} \equiv rms{d_{init}} - rms{d_{final}}$, with the profile and error bars obtained via WHAM and bootstrapping (28,30). (**B**) The O-helix angle was obtained from these five representative simulation windows labeled on the PMF curve in (A). (**C**) The distance between Y639 and RNA3′-2OH is also shown. (**D**) The five representative structures along the reaction coordinate are shown (in stereo views), corresponding to the five umbrella sampling windows (from the initial pre-insertion config 1 to the final insertion config 5). A pre-insertion configuration (config 1) with TN(i), Y639, O-helix, MgA/B, rATP and RNA 3′ denoted is also shown.

During the insertion process of rATP, the free energy rose quickly from the initial pre-insertion state (configuration or config 1) to the transition intermediate state (config 3), with an activation barrier of $\Delta E_{in}^c$∼3 ± 0.6 k_B_T (see Figure 2A). The appearance of the activation barrier correlated well with substantial O-helix opening motions (from ∼10° ± 2° at config 2 to ∼22° ± 4° at config 3, see Figure 2B). The free energy thereafter dropped, and the final insertion state (config 5) of rATP was ∼ 3 ± 0.4 k_B_T more stabilized than the initial pre-insertion state.

In the simulation, the O-helix closed well into the insertion state (from ∼8° ± 6° at config 4 to ∼5° ± 2° at config 5). Meanwhile, the Tyr639 side chain shifted away from the active site or the 3′-end of the RNA transcript (see Figure 2C), as rATP moved closely toward the 3′-end of the RNA in the insertion configuration. The rATP insertion snapshots from config 1 to 5 are seen with stereo views in Figure 2D, while the insertion dynamics can be visualized from Supplementary Movie S1.

Note that in the simulation, the two magnesium ions MgA (shown in pink in Figure 2D) and MgB (in magenta) initially stayed around sugar (3′OH) and phosphate groups of rATP, respectively. Later, MgA moved toward the 3′-end of RNA, and MgB toward in between β and γ phosphates (or the PPi part). After the catalytic reaction, MgA is supposed to remain around the 3′-end of the RNA, while MgB leaves the active site together with the PPi group release (59,60).

### Non-cognate rGTP *on-path* insertion: rGTP is less stabilized than the cognate rATP at pre-insertion *on-path* while experiences similar insertion energetics as rATP

Next, we explored the insertion energetics of the non-cognate rGTP as following the insertion dynamics *on-path*. The pre-insertion complex of rGTP *on-path* was constructed alchemically from the rATP pre-insertion complex, as reported previously (22). In particular, wobble base pairing between rGTP and template TN(i) was identified in the continuing equilibrium simulation of the pre-insertion complex (see Supplementary Figures S3A and S3C). According to the previous alchemical simulation, a relative binding free energy of rGTP vs. rATP at pre-insertion was obtained as $\Delta \Delta {G_b}$∼ 3k_B_T (22).

**Figure 3.**
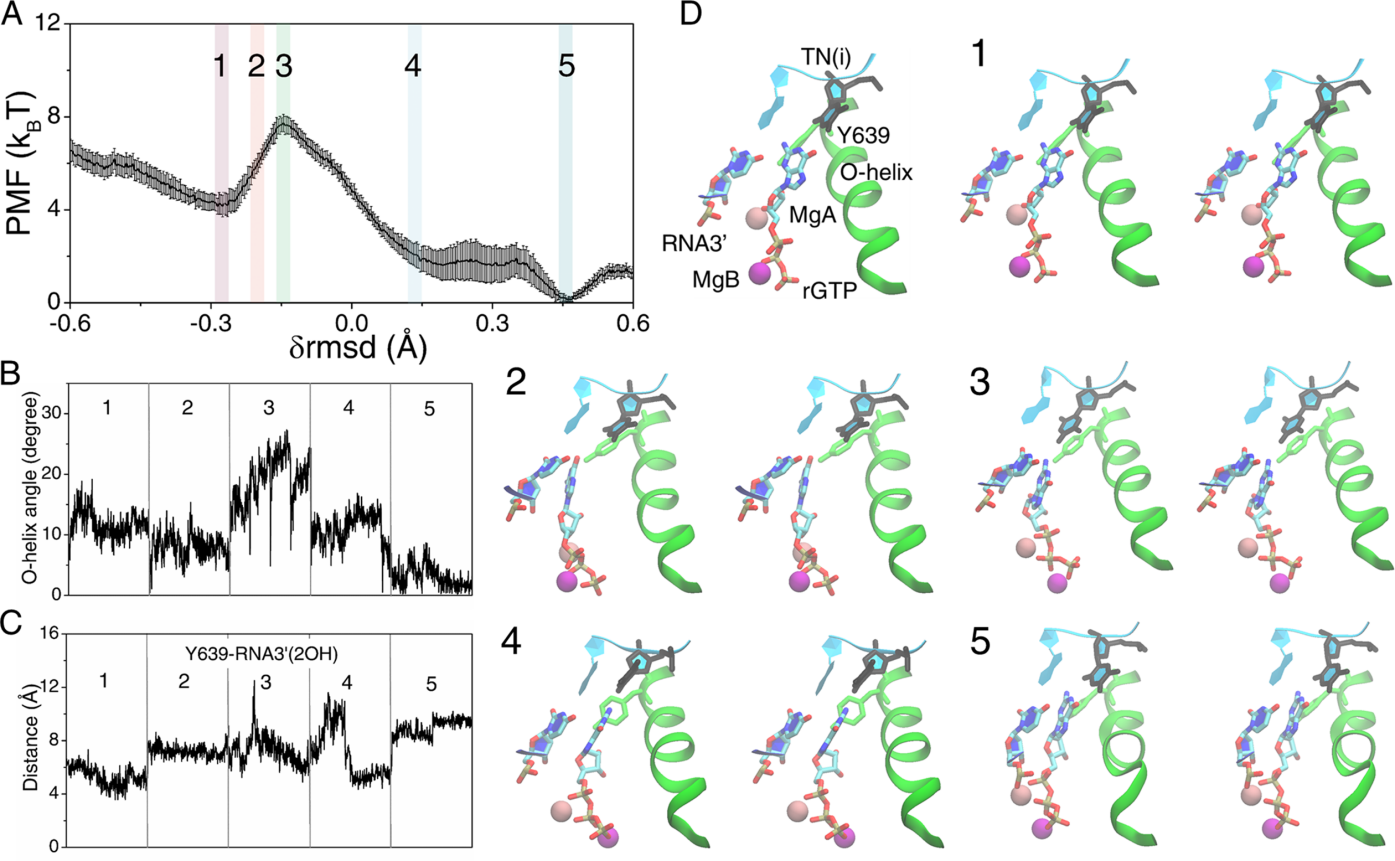
The PMF for the non-cognate rGTP bound RNAP complex from the *on-path* pre-insertion state to the insertion state and key structural measurements. (**A**) The PMF obtained from the umbrella sampling simulations along the collective reaction coordinate ${\rm{\delta rmsd}}$. (**B**) The O-helix angle obtained from the five windows labeled on the PMF curve. (**C**) The distance between Y639 and RNA3′-2OH. (**D**) The five representative structures along the reaction coordinate (in stereo views). A pre-insertion configuration (config 1) with TN(i), Y639, O-helix, MgA/B, rGTP and RNA 3′ denoted is also shown.

The PMF of the *on-path* insertion of rGTP demonstrated a similar activation barrier ($\Delta E_{in}^{on}$∼ 3.3 ± 0.2 k_B_T, see Figure 3A) as that of rATP. The O-helix also opened largely into the transition intermediate state (∼20 ± 4° at config 3, see Figure 3B) during the rGTP insertion, and eventually reached to a closed state in the insertion complex (∼5 ± 2° at config 5). Both Tyr639 and rGTP followed the similar trend as in the rATP insertion case, moving respectively away (see Figure 3C) and toward the 3′-end of the RNA. The insertion complex was energetically more stable than the *on-path* pre-insertion complex of rGTP (∼4.0 ± 0.2 k_B_T). The rGTP *on-path* insertion snapshots from config 1 to 5 are shown (in Figure 3D), and the dynamics process can be seen in Supplementary Movie S2.

### Non-cognate rGTP *off-path* insertion: The *off-path* insertion barrier of rGTP is ∼4 k_B_T higher that of the cognate rATP

Then we determined the insertion energetics of the non-cognate GTP following the *off-path* insertion dynamics. The *off-path* pre-insertion structure of rGTP was obtained by directly replacing rATP by rGTP in the crystal structure of the cognate pre-insertion complex (20,21). Notably, Tyr639 interacted closely with rGTP in the *off-path* pre-insertion complex, in order to ‘drag’ rGTP away from the *on-path* pre-insertion site (21). The corresponding distance between Tyr639 and the 3′-end RNA remained large in the equilibrium simulation of the *off-path* pre-insertion complex. Meanwhile, the template TN(i) deviated largely from rGTP at *off-path* pre-insertion (config 1).

**Figure 4.**
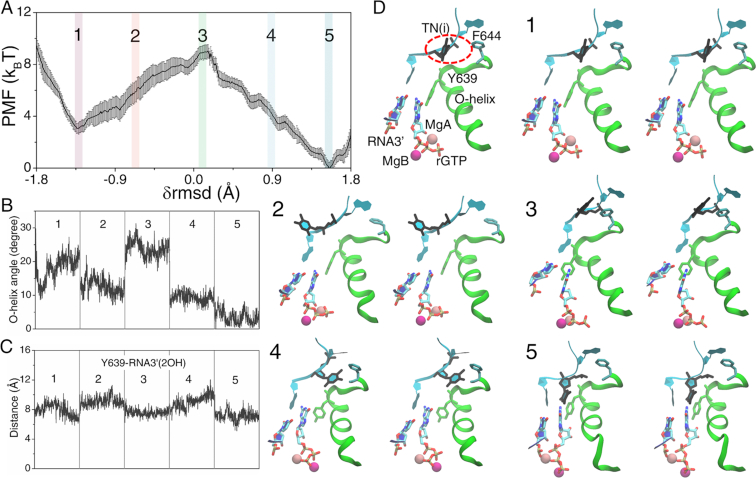
The PMF for the non-cognate rGTP bound RNAP complex from the pre-insertion *off-path* to the insertion state and key structure measures. (**A**) The PMF obtained from the umbrella sampling simulations along the collective reaction coordinate ${\rm{\delta rmsd}}$. (**B**) The O-helix angle obtained from the five windows labeled on the PMF curve. (**C**) The distance between Y639 and RNA3′-2OH. (**D**) The five representative structures (in stereo views). A pre-insertion configuration (config 1) with TN(i), Y639, O-helix, MgA/B, rGTP, and RNA 3′ denoted is also shown. The red dashed circle highlights the significant base rotation of the template TN(i).

Following the *off-path* insertion process of rGTP, the insertion barrier ($\Delta E_{in}^{off}$∼ 7.6 ± 0.6 k_B_T) turned out to be significantly larger than that of rATP or the *on-path* rGTP insertion (i.e. with an insertion selection energy $\Delta _{in}^ + \equiv \Delta E_{in}^{off} - \Delta E_{in}^c$ ∼ 4 k_B_T; see Figure 4A). Correspondingly, the O-helix opened significantly to ∼25° ± 3° in the transition intermediate state (config 3, see Figure 4B), which seemed to contribute to the large insertion barrier. Anyhow, the O-helix could still close to ∼5° ± 2° in the insertion complex (config 5). The distance between Tyr639 and the 3′-end RNA remained large till the end of the insertion process (see Figure 4C). The final *off-path* insertion structure of rGTP resembled with that of the *on-path* insertion structure, according to the local RMSD, the Tyr639-rGTP distance, and the O-helix rotation angle measurements (see Supplementary Figure S4). The rGTP *off-path* insertion snapshots from config 1 to 5 are shown in Figure 4D and the dynamic process can be seen in Supplementary Movie S3.

Notably, the template TN(i) could hardly interact or form hydrogen bonds with rGTP throughout the *off-path* insertion process. Indeed, TN(i) had its base fluctuated significantly (see Figure 4D), and the base orientation angle varied much more than that in the *on-path* insertion process (see Supplementary Figures S5A and C).

### Aligning PMFs of the non-cognate rGTP and cognate rATP: rGTP appears prohibited from *on-path* association, non-trapped at *off-path* pre-insertion, and inhibited during *off-path* insertion

**Figure 5.**
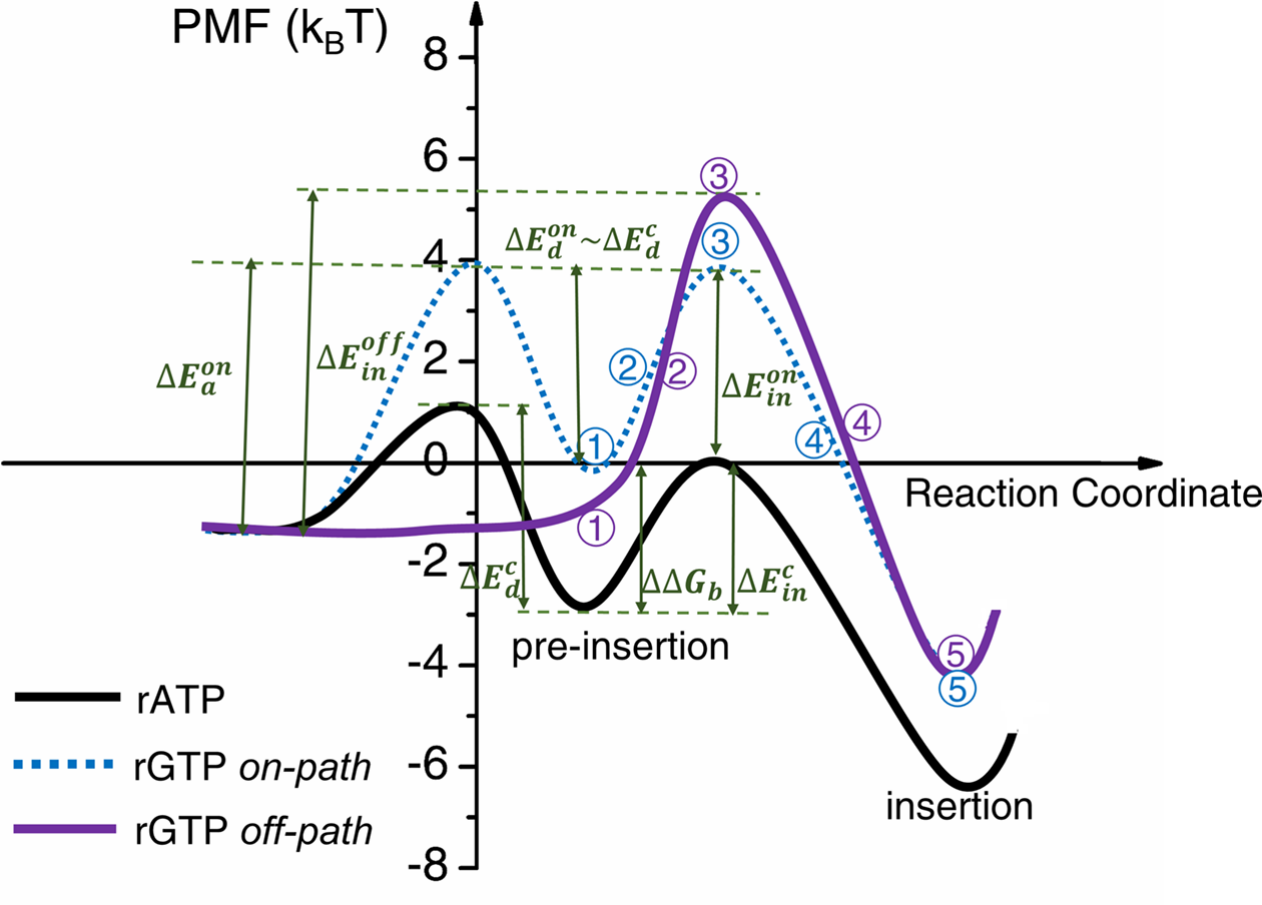
The schematic PMFs from the nucleotide pre-insertion to insertion of the cognate rATP and non-cognate rGTP. The PMF of rATP is shown in black curve, the *on-path* and *off-path* PMFs of rGTP are shown in blue (dashed line) and purple, respectively. The relative binding free energy of the *on-path* rGTP pre-insertion (config 1) to that of rATP has been evaluated alchemically (22). The final insertion configurations (config 5) of the rGTP *on-path* and *off-path* are structurally similar (see Supplementary Figure S5). The dissociation barriers at the nucleotide pre-insertion were investigated previously (61). See more in the text.

In order to obtain the selection energetics or relative free energies between the non-cognate and cognate nucleotide species, one needs to align the PMFs of rGTP and rATP together. The *on-path* PMF of the non-cognate rGTP insertion was placed above that of rATP, due to a positive relative binding free energy between rGTP and rATP at the pre-insertion site ($\Delta \Delta {G_b}$∼ 3 k_B_T) (22). Additionally, one put the rGTP *on-path* and *off-path* PMFs together by merging the two final insertion configurations that are structurally similar (see Supplementary Figure S4). This way, we aligned PMFs of both the *on-path* and *off-path* non-cognate rGTP with that of the cognate rATP, from the pre-insertion to the insertion state (see Figure 5).

In another recent simulation study of the T7 RNAP pre-insertion complex, a barrier of the nucleotide dissociation from the pre-insertion state (*III*) to the post-translocation state (*II*, see Figure 1B) was determined for the cognate rATP ($as\ \Delta E_d^c$ ∼ 4 k_B_T), while no barrier was shown for the non-cognate ones (prepared at the *off-path* configurations) (61). That says, the cognate rATP is trapped at the pre-insertion site to be prevented from dissociating, while the non-cognate rGTP *off-path* is not, as there is no dissociation barrier for the rGTP *off-path* binding (i.e., with the dissociation selection energy $\Delta _b^{-} = \Delta E_d^c - \Delta E_d^{off}\sim 4\ k_B T$). If one further assumes that the *on-path* non-cognate rGTP maintains a same dissociation barrier, or say, is similarly trapped as the cognate rATP $( {\Delta _b^ - = \Delta E_d^c - \Delta E_d^{on} \sim 0} )$, one can then provide rGTP vs rATP free energy profiles from initial binding/unbinding at pre-insertion and further to insertion, with the full schematics shown in Figure 5.

Additionally, if one assumes that both the *on-path* and *off-path* non-cognate pre-insertion complexes are quasi-equilibrated, then it can be estimated that their populations are ∼0.5% and 99.5%, respectively, due to an *on-path* association barrier $\Delta E_a^{on}$ ∼5.3 k_B_T of the mismatched rGTP obtained from the above calculations (also see Figure 5). Consequently, the *on-path* is prohibited from accessing at the very beginning of the mismatched rGTP association. Alternatively, substantial selection against the mismatched rGTP takes place *off-path* through both the dissociation at pre-insertion (i.e., no dissociation barrier or no trapping) and inhibition during insertion (high insertion barrier).

### Non-cognate dATP *on-path* insertion: The *on-path* insertion barrier of dATP is ∼3 k_B_T higher than that of the cognate rATP and two magnesium ions switch positioning during the insertion

Meanwhile, we also investigated the insertion energetics and structural dynamics of the non-cognate dATP, following its *on-path* insertion first. The *on-path* pre-insertion configuration of the dATP was also obtained alchemically from the rATP pre-insertion complex (see Materials and Methods). In the continuing equilibrium simulation of this complex, base pairing between dATP and the template TN(i) was also identified (see Supplementary Figures S3B and D). The calculated relative binding free energy between dATP and rATP at the pre-insertion binding site was $\Delta \Delta {G_b}$= –1 ± 0.2 k_B_T. The O-helix opened less in the *on-path* pre-insertion complex of dATP (∼11 ± 3°), comparing with that of the rATP.

**Figure 6.**
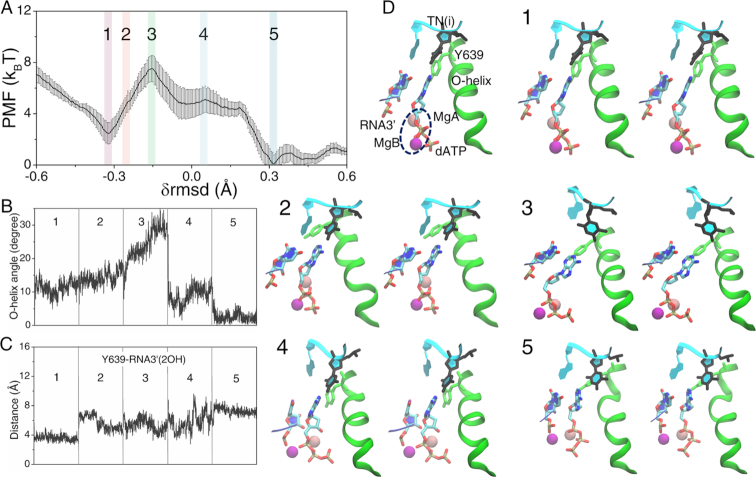
The constructed PMF for the non-cognate dATP bound structure from the pre-insertion *on-path* to the insertion state and key structure measures. (**A**) The PMF obtained from the umbrella sampling simulations along the collective reaction coordinate ${\rm{\delta rmsd}}$. (**B**) The O-helix angle obtained from the five windows labeled on the PMF curve. (**C**) The distance distribution between Y639 and RNA3′-2OH. (**D**) The five representative structures along the reaction coordinate (in stereo views). A pre-insertion configuration (config 1) with TN(i), Y639, O-helix, MgA/B, dATP and RNA 3′ denoted is also shown. The black dashed circle highlights the two magnesium ions (MgA and MgB) that switch roles in stay and leave.

The PMF of the *on-path* dATP insertion demonstrated a fairly high activation barrier ($\Delta E_{in}^{on}$∼ 6 ± 1 k_B_T, see Figure 6A), which led to $\Delta _{in}^ + \equiv \Delta E_{in}^{on} - \Delta E_{in}^c$∼ 3 k_B_T (i.e. the insertion selection energy). The resistance of the O-helix closing during the dATP insertion appeared quite strong, as the O-helix opened up above ∼30° in the transition intermediate state (see Figure 6B). In the end, the O-helix still successfully closed in the insertion complex (∼5 ± 2°). At the same time, Tyr639 and dATP also followed the similar trend as in the rATP or the *on-path* rGTP insertion case, by moving away and toward the 3′-end of the RNA, respectively (see Figure 6C). The final insertion complex of dATP was only ∼1 k_B_T more stable than the pre-insertion *on-path* configuration. The corresponding insertion snapshots from config 1 to 5 are seen in Figure 6D and the dynamics can be viewed in Supplementary Movie S4.

Interestingly, during this insertion process, two magnesium ions, MgA and MgB switched their positioning or roles for the incoming catalysis. MgA was supposed to stay close to the 3′-end of RNA before and after the catalysis, while MgB would leave along with the PPi product release after the catalysis. During the *on-path* insertion of dATP, however, MgA moved away from the sugar and toward the β phosphate soon, due to lack of a negatively charged oxygen atom on the deoxyribose sugar. Consequently, MgB was forced to move away from the β phosphate and shifted instead toward the 3′-end of RNA (see Figure 6D). Comparisons between the MgA and MgB transitions during the nucleotide insertion process for the cognate rATP, non-cognate rGTP, and dATP are summarized in Supplementary Figure S6. We repeated the simulations of the transition state config 3 and 4 for three times in the *on-path* dATP insertion process, MgA and MgB always demonstrated the above switching behaviors. Alternatively, we could reproduce the switching (see Supplementary Figure S7A&B) using the CHARMM force field (62,63) via either SwissParam (64) or CCenFF (65,66) for the force field generation. Furthermore, when we locally refined group charges of (dATP & MgB) and (rATP & MgB), respectively (67) (see RESP charges in Supplementary Tables S1 and S2), still in the AMBER force field (37), the switching also persisted in the dATP *on-path* simulation but not the rATP case (see Supplementary Figure S7C). Hence, the two magnesium ion switching appeared to be robust in the dATP *on-path* insertion simulation, regardless some variations of the force field treatments.

### Non-cognate dATP *off-path*: The *off-path* insertion barrier of dATP is slightly larger than that of rATP while the insertion state cannot be well reached

Last, we surveyed the insertion energetics and dynamics of the non-cognate dATP *off-path*. The *off-path* pre-insertion structure of dATP was constructed by directly replacing rATP by dATP in the crystal structure of the pre-insertion complex and then performing the equilibrium simulation. In the constructed *off-path* pre-insertion complex of dATP, it had been reported that Tyr639 could stack nicely with the end base pair of the DNA-RNA hybrid, while mimicking ‘base pairing’ with the pre-insertion dATP (21).

**Figure 7.**
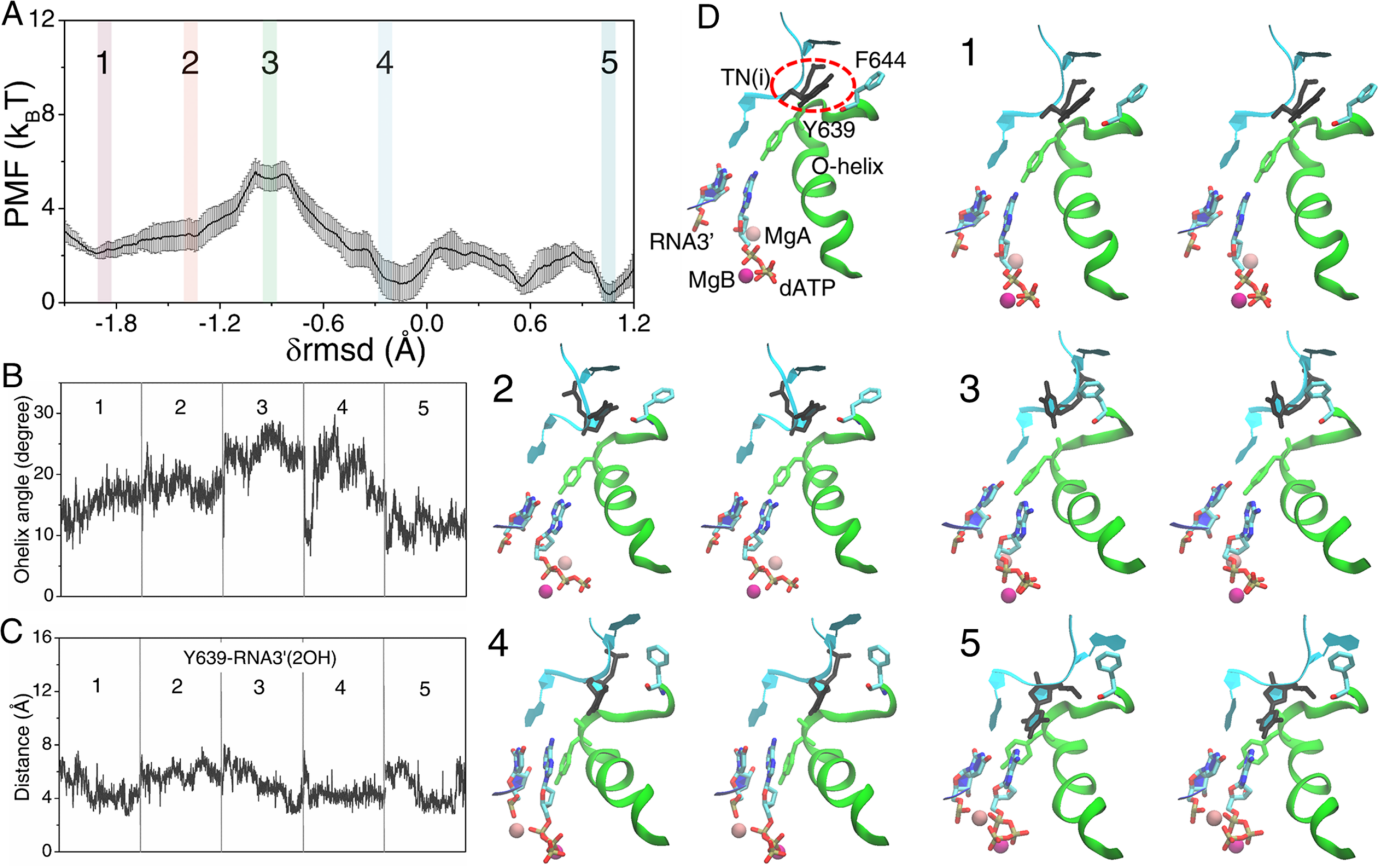
The constructed PMF of the non-cognate dATP bound structure from the pre-insertion *off-path* configuration to the semi-insertion state and key structure measures. (**A**) The PMF obtained from the umbrella sampling simulations along the collective reaction coordinate ${\rm{\delta rmsd}}$. (**B**) The O-helix angle obtained from the five windows labeled on the PMF curve. (**C**) The distance between Y639 and RNA3′-2OH. (**D**) The five representative structures along the reaction coordinate (in stereo views). A pre-insertion configuration (config 1) with TN(i), Y639, O-helix, MgA/B, dATP, and RNA 3′ denoted is also shown. The red dashed circle highlights the significant shifting of the template TN(i) backward.

The insertion barrier ($\Delta E_{in}^{off}$∼ 4 ± 0.8 k_B_T, see Figure 7A) for the *off-path* dATP insertion was only slightly larger than that of the rATP (with the insertion selection energy$\Delta _{in}^ + \equiv \Delta E_{in}^{off} - \Delta E_{in}^c$∼1 k_B_T), or ∼2 k_B_T smaller than that of the *on-path* dATP insertion. The O-helix still opened largely (∼ 25° ± 2°) to resist the closing in the transition state, as in other cases. However, the O-helix could not successfully close below ∼10° into the ‘insertion’ complex (see config 5 in Figure 7B). Indeed, Y639 interacted closely with the RNA 3′-end (-2OH group) and remained around the active site (see Figure 7C), thus hindering the O-helix closing. Further examinations indicated that the final *off-path* insertion complex (config 5) of dATP was likely located in between config 4 and config 5 of the *on-path* dATP insertion (see Supplementary Figure S8). The dATP *off-path* insertion snapshots from config 1 to 5 are seen in Figure 7D while the dynamics can be viewed in Supplementary Movie S5.

Notably, the template TN(i) in the *off-path* dATP pre-insertion configuration had been ‘pushed’ back toward an intermediate configuration as that during the translocation (see Figure 7D config 1), and the base fluctuated significantly during the insertion process (see Supplementary Figure S5). Since the RNAP complex drifted away from the post-translocation state and adopted an intermediate configuration in between the pre- and post-translocation state (see Supplementary Figure S9), the *off-path* dATP pre-insertion complex was supposed to be slightly less stable in energetics than a post-translocation complex.

### Aligning PMFs of non-cognate dATP and cognate rATP: dATP can be repelled at the *off-path* pre-insertion and inhibited during the *on-path* insertion

**Figure 8.**
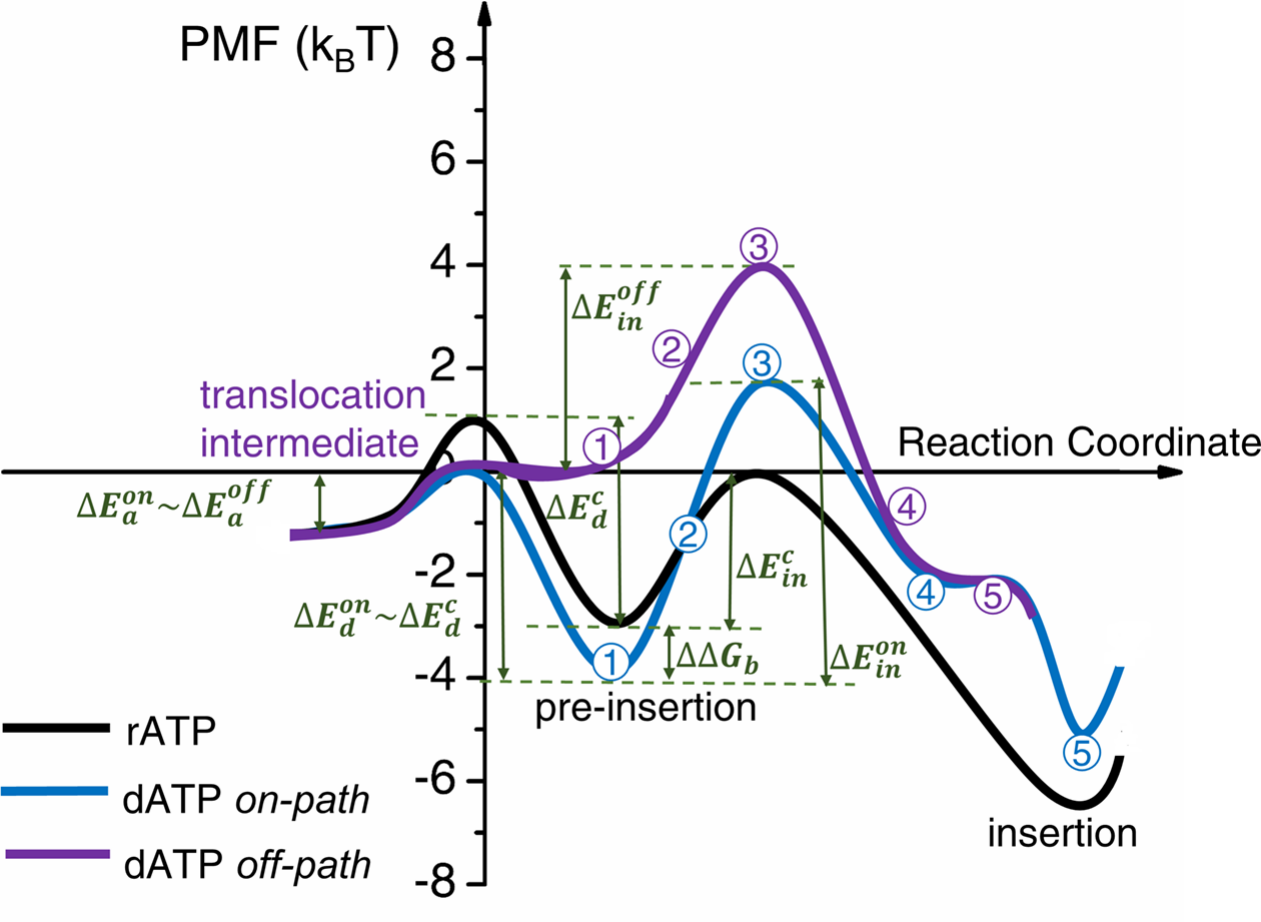
The schematic PMFs from the nucleotide pre-insertion to insertion for the cognate rATP and non-cognate dATP. The PMF of rATP is shown in black curve, the *on-path* and *off-path* PMFs of dATP are shown in blue and purple, respectively. The relative binding free energy for the *on-path* dATP pre-insertion (config 1) to that of rATP has been calculated alchemically. The final configuration (config 5) of dATP *off-path* locates in between config 4 and 5 *on-path* (see Supplementary Figure S8). The dissociation barriers at nucleotide pre-insertion are treated similarly as in Figure 5, except for the *off-path* pre-insertion being an intermediate configuration as during the translocation (see text).

Finally, we could align the PMFs of the *on- and off-path* insertion of dATP together with that of the cognate rATP, by first placing the *on-path* pre-insertion config 1 slightly below that of the rATP, and then placing the dATP *off-path* insertion config 5 in between the *on-path* config 4 and 5 (see Figure 8).

Note that we have also assumed that the dATP *on-path* dissociation from the pre-insertion site maintains a same barrier as that in the cognate rATP case, i.e. $\Delta E_d^{on}\sim\Delta E_d^c$ = 4 k_B_T, while for the dATP *off-path*, there is still no dATP trapping due to lack of a dissociation barrier $\Delta E_d^{off}$= 0, as in the *off-path* rGTP case. In addition, since the dATP *off-path* pre-insertion complex had the template TN(i) shifted backward to a translocation intermediate configuration, it should be energetically less stable than the post-translocation state, prior to the NTP binding. Furthermore, we have assumed that the translocation intermediate is energetically comparable or similar to the binding intermediate for the *on-path* dATP pre-insertion, both of which can be marginally or thermally (∼1 to 2 k_B_T) less stable than the post-translocation complex. The complete schematics on the pre-insertion to insertion PMFs are accordingly shown in Figure 8, for both the *on-* and *off-path* insertion of dATP and insertion of rATP.

Under the quasi-equilibrium assumption of the dATP binding at pre-insertion, there would be about equal probabilities for dATP accessing *on-path* and *off-path*. That says, both the *on-path* and *off-path* non-cognate insertion and selection work for the deoxy-ribonucleotide. The *on-path* selection proceeds through the insertion inhibition barrier, while the *off-path* selection works via the enhanced nucleotide dissociation at pre-insertion. According to the results presented in both Figures 5 and 8, we obtained the stepwise selection free energetics from pre-insertion to insertion (see METHODS), and inferred that into the catalytic stage $( {\Delta _c^ + \sim 7{k_B}T\,{\rm{for}}\,{\rm{rGTP}}\,{\rm{and}}\,\Delta _c^ + \cong 0\,{\rm{for}}\,{\rm{dATP}}} )$, which were summarized in the end of Supplementary Material (see Supplementary Table S3 for the selection free energetics).

## DISCUSSION

In this work, we performed atomistic simulations over microseconds to construct the PMFs of cognate and non-cognate nucleotide insertion during T7 RNAP elongation, using the umbrella sampling method following a one-dimensional collective coordinate, that presumably captures the most essential conformation changes of a highly relevant set of atoms involved in the nucleotide insertion. Together with other simulations at the nucleotide pre-insertion site to measure the relative binding free energies and nucleotide dissociation energies, we were able to determine the complete free energy profiles starting from the nucleotide pre-insertion to the insertion prior to the catalytic reaction, for both the cognate and non-cognate nucleotide species. Accordingly, the nucleotide selection energetics against the non-cognate species along the insertion paths were obtained, for either rejection backward or inhibition forward on the reaction path, at both the pre-insertion and insertion checkpoints.

We recognize first the common features revealed for the non-cognate nucleotides at the *off-path* pre-insertion configuration, which serves as the initial screening or kinetic checkpoint of the nucleotide selection. In contrast with a significant dissociation barrier (∼4 k_B_T) that traps the cognate nucleotide at the pre-insertion site, easy dissociations of the *off-path* non-cognate species let go a large portion of the erroneous nucleotide species, leading to an error rate down to ∼10^−2^. At the next selection checkpoint starting from the pre-insertion to the insertion state along the *off-path*, the mismatched rGTP leaked from the initial screening can be further kinetically hindered by an enhanced insertion free energy barrier, which is significantly higher (∼4 k_B_T) than that of the cognate species and thus reduces the error rate down to ∼10^−3^. Meanwhile, although the *on-path* insertion energetics of the mismatched rGTP turns out to be similar to that of the cognate species, rarely rGTP can be loaded *on-path* at the pre-insertion entrance (<1% of the rGTP population, with the *on-path* error rate cut below 10^−2^ as well), thus the *on-path* is almost non-accessible to the mismatch nucleotide, so that it does contribute further to the non-cognate nucleotide insertion and subsequent selection.

In comparison, the non-cognate deoxy-ribonucleotide dATP, accessible comparably to both the *on-path* and *off-path* insertion processes, is hindered over the cognate species during the *on-path* insertion (error rate down to ∼10^−2^). Interestingly, the *on-path* insertion of dATP seems to involve two-magnesium-ion switching their roles during the nucleotide insertion. Along the *off-path* insertion of dATP, however, after the initial screening or rejection at pre-insertion, no such an enhanced insertion barrier shows, yet only a semi-insertion state can be reached. Upon the complete insertion, the error rate of dATP over rATP drops to the experimentally identified value (∼10^−2^), and no further selection seems to be needed into the catalytic stage. In contrast, to achieve an elongation error rate ∼10^−4^, we inferred that the mismatched rGTP needs to be further selected by a catalytic barrier significantly higher (∼7 k_B_T) than that of the cognate one.

### The non-cognate nucleotide is initially screened at the pre-insertion site for an *off-path* rejection, coordinated by Tyr639 and template TN(i) motions

From previous studies, we recognize that an *off-path* binding or pre-insertion configuration of the non-cognate nucleotide exists, no matter for the base-mismatch or sugar-deficient incoming NTPs (21,22). In particular, Tyr639 has been found to play a critical role in the nucleotide substrate differentiation or selection, both experimentally and computationally, e.g. as Y639F mutation weakens the deoxy group detection and leads to comparable recruitments of both dNTP and rNTP (21,23,24,68). At the post-translocation state prior to the nucleotide binding, the O-helix on the fingers subdomain opens, Tyr639 on the C-terminus of the O-helix inserts its side chain next to the pre-insertion site (20,32). Consequently, Tyr639 interacts closely with the incoming NTP for pre-insertion, e.g. by grabbing on the non-cognate species or even mimicking ‘base paring’ with the dNTP, as being previously found (21). It seems that Tyr639 competes with the template TN(i) for the incoming NTP interaction, and it is, of course, the template TN(i) that essentially determines a cognate or a non-cognate species incoming. In our current simulation, we see that TN(i) deviates away significantly upon the *off-path* non-cognate rGTP/dATP pre-insertion. In particular, TN(i) rotates its side chain away from the rGTP base, or in the dATP case, TN(i) even drifts backward to an intermediate position as during the translocation. That says, the post-translocation complex of T7 RNAP is sensitive enough to respond quickly to the incoming non-cognate NTP, by utilizing an *off-path* binding and filtering strategy.

Another simulation study then shows that the non-cognate nucleotide at pre-insertion, prepared at the *off-path* configuration, easily dissociates from the RNAP binding site without free energy hindrance, while the cognate nucleotide binding into the pre-insertion site is trapped by a substantial dissociation barrier ∼4 k_B_T (61). According to the stepwise kinetic model on the polymerase nucleotide selection (6), the difference of the dissociation barrier between the cognate and non-cognate species attributes exactly to the initial selection, so that to repel the non-cognate nucleotide much faster than the cognate substrate species to dissociate from the binding/pre-insertion site. By applying only this amount of initial selection, the error rate soon drops to ∼10^−2^, and can drop further upon subsequent selections during the nucleotide insertion and catalytic incorporation.

### Prohibited from the *on-path* association, the mismatched rGTP is subject to substantial inhibition during the *off-path* insertion

After the initial nucleotide screening at pre-insertion, it is still possible for some of the non-cognate nucleotides to proceed to the insertion stage of the NAC. In the case of the mismatched rGTP, we obtained an *off-path* insertion barrier ∼4 k_B_T higher than that of the cognate rATP. The high barrier coincides with the O-helix opening motion up to ∼30° in the transition state, which appears to resist the insertion. The rotational degree of the O-helix essentially links with the positioning of its C-terminal residue Tyr639. As pointed above, in the O-helix opening configuration, the side chain of Tyr639 occupies the active site to compete with the template TN(i) to associate with the incoming rGTP. As the highly fluctuating TN(i) rotates its base far away from the active site, Tyr639 successfully hinders the TN(i) base from coming close (i.e., to contribute the rGTP insertion energy barrier). In the end, Tyr639 moves away from the active site to allow the nucleotide insertion. Accordingly, the O-helix initially opens and then closes finally to coordinate with the Tyr639 motions.

Meanwhile, one sees that the entry to the *on-path* insertion is highly prohibited for the mismatched rGTP. Firstly, we have found that the *on-path* pre-insertion complex of rGTP is ∼3 k_B_T less stable than the cognate rATP complex, according to the alchemical simulation starting from the equilibrated pre-insertion complex of rATP (22). Secondly, by inspecting the structural feature such as the wobble base pairing of the *on-path* pre-insertion complex of rGTP, we assume that once the rGTP ‘squeezes’ into the *on-path* entry, there is a dissociation barrier to prevent it from unbinding, similarly as that for the cognate rATP. Consistently, we notice that further insertion of rGTP *on-path* does not incur much different energetics from the cognate insertion. It should be noted that in the MD simulation, an initial replacement of rATP by rGTP in the crystal structure of the pre-insertion complex always leads to the comparatively stable or accessible *off-path* binding configuration rather than the *on-path* one. Indeed, it is estimated that <1% population of the incoming rGTP at pre-insertion can be loaded *on-path*, due to an activation barrier derived ∼5 k_B_T for such nucleotide association.

### Aside from the *off-path* screening at pre-insertion, the non-cognate dATP can be loaded *on-path* but with insertion inhibition

In comparison, the sugar-deficient dATP can be recruited equally well to both the *on-path* and the *off-path* configurations at pre-insertion. According to alchemical calculations, the dATP *on-path* pre-insertion complex appears similarly or even slightly more stabilized than the rATP pre-insertion complex (∼1 k_B_T). By assuming as well that the dissociation barrier for the *on-path* dATP is similar to that of rATP at pre-insertion, one sees a fairly low activation barrier about 1–2 k_B_T for the dATP binding *on-path*. Meanwhile, the *off-path* pre-insertion complex of dATP demonstrates structural characters of a translocation intermediate state, in which the template TN(i) is repelled backward (69). During the Brownian alike movements of the RNAP translocation, it is expected that the intermediate energetics is marginally higher (1–2 k_B_T) than the pre and post-translocation states. Accordingly, the *off-path* pre-insertion of dATP can be thermally activated, similarly as the *on-path* pre-insertion of dATP. Therefore, there appear to be similar or say equal chances of dATP to bind *on-path* and *off-path*. Once bound into the *on-path* pre-insertion configuration, dATP indeed forms nice WC base pairing with the template TN(i). Nevertheless, an enhanced insertion barrier thereafter prevents the *on-path* dATP from inserting easily as the cognate rATP. Accordingly, both the easy dissociation at the *off-path* pre-insertion and the inhibition during the *on-path* insertion contribute to the RNAP nucleotide selectivity to be against the deoxy-ribonucleotides.

Interestingly, we notice that during the *on-path* insertion of dATP, two magnesium ions switch their roles of leave and stay. According to an united two-metal-ion mechanism in polymerase functioning, there are two magnesium ions involved in the active center reaction (59,60), with one proposed to retain permanently (‘stay’), while the other recruited *ad hoc* with the incoming NTP and gone with the releasing product PPi (‘leave’). In current work, except for the dATP system, MgA as the one stays, associates closely around the sugar (3′-OH) of the pre-insertion NTP, then moves via an intermediate configuration around α or β phosphate (around Asp537 and Asp812 as well), finally ends up being close to the 3′-end of RNA to support catalysis; MgB as the one leaves, starts closely around the β phosphate of the pre-insertion NTP, ends up fluctuating between the β and γ phosphate groups (see Supplementary Figure S6). In the dATP *on-path* case, however, due to lack of an oxygen atom on the 2′-OH of sugar, MgA very soon moves away and toward the β phosphate to ‘push’ MgB toward the 3′-end RNA, so that MgA and MgB switch their positioning in the end. Though the force field description of magnesium is still to be improved to satisfactorily address variable issues, currently observed switching events can be repeated and reproduced under various force field implementation settings (see Supplementary Figure S7). Hence, our studies suggest that magnesium ions can play special roles in the transcription fidelity control, particularly in discriminating the non-cognate dNTP from the cognate rNTP during the *on-path* insertion process. Note that along the *off-path* insertion of dATP, MgA remains far from 3′-end RNA, as the corresponding ‘insertion’ state is not quite much reached.

### To achieve an error rate sufficiently low, the mismatched rGTP needs to be further selected against during the catalytic reaction

According to biochemical measurements on the T7 RNAP transcription elongation, the error rate of replacing rATP by rGTP is about 10^−4^, while other base mismatch types can be scrutinized even more stringently (10^−5^ to 10^−6^) (11). In comparison, the error rate of a cognate rNTP replaced by dNTP of the same base appears higher at ∼10^−2^ (24). Using the selection energetics from the pre-insertion to insertion, obtained from our atomistic MD simulations and calculations (see Supplementary Table S3), we were able to derive the error rates via the chemical master equation approach (6,26). In the case of rGTP, substantial selection from the *off-path* pre-insertion rejection (∼4 k_B_T) together with the insertion inhibition (∼4 k_B_T) reduce the error rate to ∼ 10^−3^, while the *on-path* recruitment of less than 1% rGTP population also ensures the error rate to approach ∼ 10^−3^. To further reduce the error rate to 10^−4^, rGTP needs to be selected again during the catalytic reaction, which we cannot simulate using the classic MD. Notably, we can still infer that the activation barrier of catalytically adding rGTP needs to be ∼7 k_B_T higher than that of adding rATP in order to achieve an error rate at ∼10^−4^. In contrast, since the selection energetics against dATP from the pre-insertion to insertion already leads to an error rate of ∼10^−2^, it is reasonable that no further selection is needed, and the catalytic addition of dATP to the existing RNA strand, once it is fully inserted into the active site, can be as easy as the cognate rATP.

Note that in general the catalytic checkpoint works more or less as the catalytic rate of an inserted non-cognate nucleotide is commonly expected to be lower than that of the cognate one, due to non-satisfactory insertion configuration at the active site in the absence of the WC base pairing. For example, for T7 DNAP the experimentally detected catalytic rates of cognate and non-cognate species are 360 and 0.3 s^−1^ (16), respectively. For a bacterial multi-subunit RNAP, the cognate and non-cognate catalytic rates can be 100 and ∼10^−2^ s^−1^ (2), respectively. It has also been pointed out that the trigger loop in the multi-subunit RNAP acts as a kinetic selector for correct NTPs, functioning analogously to the fingers subdomains in the single-subunit polymerases by promoting catalysis of correct NTPs efficiently but incorrect substrates inefficiently (8).

From our previous study (6), we recognize that a same amount of selection energy arising at an early checkpoint would lead to a lower error rate than that being achieved late on the reaction path. For example, an overall selection energy of ∼8 k_B_T distributed from the pre-insertion checkpoint (∼ 4 k_B_T) toward the insertion checkpoint (∼4 k_B_T) can lead to an error rate down to ∼ 10^−3^. In comparison, a significant amount of selection energy (∼7 k_B_T) is still required toward the late catalytic checkpoint to only reduce the error rate for one order of magnitude, i.e. from 10^−3^ to 10^−4^. Accordingly, in order to achieve high fidelity via the stepwise substrate selection, say for a limited total amount of selection energetics, it is advantageous to select as early as possible on the reaction path. In current study of the viral T7 RNAP, it appears that the nucleotide selections conducted from the pre-insertion to the insertion work substantially to reduce the error rate close to the desired magnitude. The catalytic step serves for the base selection as well in T7 RNAP, but not necessarily for a significant improvement on the error rate reduction. The predicted catalytic selection energetics for the base-mismatch incorporation (e.g. rG replacing rA) can be examined in further experimental and computational studies.

Accordingly, one expects that low fidelity RNAPs in some circumstances skip nucleotide selections at early checkpoints until toward the late catalytic stage or to the end of the NAC. In contrast, for high transcription fidelity control, the pre-chemical steps such the O-helix linked fingers subdomain closing in the single-subunit polymerases need to play an essential role, so that non-cognate nucleotides have already been strongly selected against from the initial association to the full insertion stage, prior to the catalytic reaction.

## CONCLUSION

According to atomistic MD simulations and free energy calculations, we have demonstrated that the viral T7 RNAP conducts nucleotide screening and selection substantially starting from the nucleotide pre-insertion: by initially rejecting the non-cognate species upon an *off-path* association while trapping the cognate species *on-path* for the WC base pairing, and by further inhibiting the non-cognate nucleotide during insertion. An error rate ∼ 10^−3^ can be achieved pre-chemically for the mismatched rGTP, which mainly binds and inserts *off-path* while being prohibited from accessing *on-path*. To achieve an error rate ∼10^−4^ or lower, further selection during catalytic reaction seems to be required to be against the base mismatch species. In comparison, the sugar deficient dATP is either readily rejected upon the *off-path* pre-insertion, or it accesses *on-path* while experiencing insertion inhibition, likely also under the coordination of two magnesium ions. An error rate ∼10^−2^ is achieved to prevent the dATP incorporation over the cognate rATP, without further differentiation into the catalytic stage.

## Supplementary Material

Supplementary Data

## References

[1] Sydow J.F., Cramer P. RNA polymerase fidelity and transcriptional proofreading. Curr. Opin. Struct. Biol. 2009; 19:732–739. 19914059 10.1016/j.sbi.2009.10.009

[2] Yuzenkova Y., Bochkareva A., Tadigotla V.R., Roghanian M., Zorov S., Severinov K., Zenkin N. Stepwise mechanism for transcription fidelity. BMC Biol. 2010; 8:54. 20459653 10.1186/1741-7007-8-54 PMC2874521

[3] Moustafa I.M., Shen H., Morton B., Colina C.M., Cameron C.E. Molecular dynamics simulations of viral RNA polymerases link conserved and correlated motions of functional elements to fidelity. J. Mol. Biol. 2011; 410:159–181. 21575642 10.1016/j.jmb.2011.04.078 PMC3114172

[4] Kellinger M.W., Ulrich S., Chong J., Kool E.T., Wang D. Dissecting chemical interactions governing RNA polymerase II transcriptional fidelity. J. Am. Chem. Soc. 2012; 134:8231–8240. 22509745 10.1021/ja302077d PMC3367139

[5] Shen H., Li G. Bridging the missing link between structure and fidelity of the RNAdependent RNA polymerase from Poliovirus through free energy simulations. J. Chem. Theory Comput. 2014; 10:5195–5202. 26584391 10.1021/ct5006449

[6] Yu J. Eflcient fidelity control by stepwise nucleotide selection in polymerase elongation. Mol. Math. Biol. 2014; 2:141–160.

[7] Wang B., Opron K., Burton Z.F., Cukier R.I., Feig M. Five checkpoints maintaining the fidelity of transcription by RNA polymerases in structural and energetic details. Nucleic Acids Res. 2015; 43:1133–1146. 25550432 10.1093/nar/gku1370 PMC4333413

[8] Kaplan C.D. The architecture of RNA polymerase fidelity. BMC Biol. 2010; 8:85. 20598112 10.1186/1741-7007-8-85 PMC2889878

[9] McCulloch S.D., Kunkel T.A. The fidelity of DNA synthesis by eukaryotic replicative and translesion synthesis polymerases. Cell Res. 2008; 18:148. 18166979 10.1038/cr.2008.4 PMC3639319

[10] Huang J., Brieba L.G., Sousa R. Misincorporation by Wild-Type and mutant T7 RNA Polymerases: Identification of interactions that reduce misincorporation rates by stabilizing the catalytically incompetent open conformation. Biochemistry. 2000; 39:11571–11580. 10995224 10.1021/bi000579d

[11] Sultana S., Solotchi M., Ramachandran A., Patel S.S. Transcriptional fidelities of human mitochondrial POLRMT, yeast mitochondrial Rpo41, and Phage T7 single-subunit RNA polymerases. J. Biol. Chem. 2017; 292:18145–18160. 28882896 10.1074/jbc.M117.797480 PMC5672038

[12] Sousa R. Structural and mechanistic relationships between nucleic acid polymerases. Trends Biochem. Sci. 1996; 21:186–190. 8871404

[13] Steitz T.A. DNA Polymerases: Structural diversity and common mechanisms. J. Biol. Chem. 1999; 274:17395–17398. 10364165 10.1074/jbc.274.25.17395

[14] Cramer P. Common structural features of nucleic acid polymerases. BioEssays. 2002; 24:724–729. 12210533 10.1002/bies.10127

[15] Yu J. Computational investigations on polymerase actions in gene transcription and replication: Combining physical modeling and atomistic simulations. Chinese Phys. B. 2016; 25:018706.

[16] Johnson K.A. The kinetic and chemical mechanism of high-fidelity DNA polymerases. Biochim. Biophys. Acta (BBA)-Proteins Proteomics. 2010; 1804:1041–1048. 20079883 10.1016/j.bbapap.2010.01.006 PMC3047511

[17] Schlick T., Arora K., Beard W.A., Wilson S.H. Perspective: pre-chemistry conformational changes in DNA polymerase mechanisms. Theor. Chem. Acc. 2012; 131:1287. 23459563 10.1007/s00214-012-1287-7 PMC3583561

[18] Wu E.Y., Beese L.S. The structure of a high fidelity DNA polymerase bound to a mismatched nucleotide reveals an “Ajar” intermediate conformation in the nucleotide selection mechanism. J. Biol. Chem. 2011; 286:19758–19767. 21454515 10.1074/jbc.M110.191130 PMC3103354

[19] Miller B.R. III, Parish C.A., Wu E.Y. Molecular dynamics study of the opening mechanism for DNA Polymerase I. PLos Comput. Biol. 2014; 10:e1003961. 25474643 10.1371/journal.pcbi.1003961 PMC4256020

[20] Temiakov D., Patlan V., Anikin M., McAllister W.T., Yokoyama S., Vassylyev D.G. Structural basis for substrate selection by T7 RNA polymerase. Cell. 2004; 116:381–391. 15016373 10.1016/s0092-8674(04)00059-5

[21] Duan B., Wu S., Da L.-T., Yu J. A critical residue selectively recruits nucleotides for T7 RNA polymerase transcription fidelity control. Biophys. J. 2014; 107:2130–2140. 25418098 10.1016/j.bpj.2014.09.038 PMC4223216

[22] E C., Duan B., Yu J. Nucleotide selectivity at a preinsertion checkpoint of T7 RNA polymerase transcription elongation. J. Phys. Chem. B. 2017; 121:3777–3786. 28199109 10.1021/acs.jpcb.6b11668

[23] Sousa R., Padilla R. A mutant T7 RNA polymerase as a DNA polymerase. EMBO J. 1995; 14:4609–4621. 7556104 10.1002/j.1460-2075.1995.tb00140.x PMC394553

[24] Brieba L.G., Sousa R. Roles of histidine 784 and tyrosine 639 in ribose discrimination by T7 RNA polymerase. Biochemistry. 2000; 39:919–923. 10653635 10.1021/bi992324+

[25] Anand V.S., Patel S.S. Transient state kinetics of transcription elongation by T7 RNA polymerase. J. Biol. Chem. 2006; 281:35677–35685. 17005565 10.1074/jbc.M608180200

[26] Long C., Yu J. Balancing Non-Equilibrium driving with nucleotide selectivity at kinetic checkpoints in polymerase fidelity control. Entropy. 2018; 20:306. 10.3390/e20040306 PMC7512825 33265397

[27] Torrie G.M., Valleau J.P. Nonphysical sampling distributions in Monte Carlo free-energy estimation: Umbrella sampling. J. Comput. Phys. 1977; 23:187–199.

[28] Kumar S., Rosenberg J.M., Bouzida D., Swendsen R.H., Kollman P.A. THE weighted histogram analysis method for free‐energy calculations on biomolecules. I. The method. J. Comput. Chem. 1992; 13:1011–1021.

[29] Kastner J. Umbrella Sampling. WIREs Comput. Mol. Sci. 2011; 1:932–942.

[30] Grossfield A. WHAM: the weighted histogram analysis method. 2012; version 2.06.

[31] Gillespie D.T. A rigorous derivation of the chemical master equation. Physica A. 1992; 188:404–425.

[32] Yin Y.W., Steitz T.A. The structural mechanism of translocation and helicase activity in T7 RNA polymerase. Cell. 2004; 116:393–404. 15016374 10.1016/s0092-8674(04)00120-5

[33] Yin Y.W., Steitz T.A. Structural basis for the transition from initiation to elongation transcription in T7 RNA polymerase. Science. 2002; 298:1387–1395. 12242451 10.1126/science.1077464

[34] Berendsen H.J., van der Spoel D., van Drunen R. GROMACS: a message-passing parallel molecular dynamics implementation. Comput. Phys. Commun. 1995; 91:43–56.

[35] Abraham M.J., van der Spoel D., Lindahl E., Hess B. the GROMACS Development Team GROMACS User Manual. 2016; version 5.1.2 www.gromacs.org.

[36] Guy A.T., Piggot T.J., Khalid S. Single-stranded DNA within nanopores: conformational dynamics and implications for sequencing; a molecular dynamics simulation study. Biophys. J. 2012; 103:1028–1036. 23009852 10.1016/j.bpj.2012.08.012 PMC3433622

[37] Hornak V., Abel R., Okur A., Strockbine B., Roitberg A., Simmerling C. Comparison of multiple Amber force fields and development of improved protein backbone parameters. Proteins: Struct. Funct. Bioinformatics. 2006; 65:712–725. 10.1002/prot.21123 PMC4805110 16981200

[38] Joung I.S., Cheatham T.E. III Determination of alkali and halide monovalent ion parameters for use in explicitly solvated biomolecular simulations. J. Phys. Chem. B. 2008; 112:9020–9041. 18593145 10.1021/jp8001614 PMC2652252

[39] Joung I.S., Cheatham T.E. III Molecular dynamics simulations of the dynamic and energetic properties of alkali and halide ions using water-model-specific ion parameters. J. Phys. Chem. B. 2009; 113:13279–13290. 19757835 10.1021/jp902584c PMC2755304

[40] Meagher K.L., Redman L.T., Carlson H.A. Development of polyphosphate parameters for use with the AMBER force field. J. Comput. Chem. 2003; 24:1016–1025. 12759902 10.1002/jcc.10262

[41] Price D.J., Brooks C.L. III A modified TIP3P water potential for simulation with Ewald summation. J. Chem. Phys. 2004; 121:10096–10103. 15549884 10.1063/1.1808117

[42] Darden T., York D., Pedersen L. Particle mesh Ewald: An N⋅ log (N) method for Ewald sums in large systems. J. Chem. Phys. 1993; 98:10089–10092.

[43] Essmann U., Perera L., Berkowitz M.L., Darden T., Lee H., Pedersen L.G. A smooth particle mesh Ewald method. J. Chem. Phys. 1995; 103:8577–8593.

[44] Parrinello M., Rahman A. Polymorphic transitions in single crystals: a new molecular dynamics method. J. Appl. Phys. 1981; 52:7182–7190.

[45] Nosé S., Klein M. Constant pressure molecular dynamics for molecular systems. Mol. Phys. 1983; 50:1055–1076.

[46] Bussi G., Donadio D., Parrinello M. Canonical sampling through velocity rescaling. J. Chem. Phys. 2007; 126:014101. 17212484 10.1063/1.2408420

[47] Torrie G.M., Valleau J.P. Monte Carlo free energy estimates using non-Boltzmann sampling: application to the sub-critical Lennard-Jones fluid. Chem. Phys. Lett. 1974; 28:578–581.

[48] Weiss D.R., Levitt M. Can morphing methods predict intermediate structures. J. Mol. Biol. 2009; 385:665–674. 18996395 10.1016/j.jmb.2008.10.064 PMC2691871

[49] Banavali N.K., Roux B. Free energy landscape of A-DNA to B-DNA conversion in aqueous solution. J. Ame. Chem. Soc. 2005; 127:6866–6876. 10.1021/ja050482k 15869310

[50] Arora K., Brooks C.L. Large-scale allosteric conformational transitions of adenylate kinase appear to involve a population-shift mechanism. Proc. Natl. Acad. Sci. U.S.A. 2007; 104:18496–18501. 18000050 10.1073/pnas.0706443104 PMC2141805

[51] Tribello G.A., Bonomi M., Branduardi D., Camilloni C., Bussi G. PLUMED 2: new feathers for an old bird. Comput. Phys. Commun. 2014; 185:604–613.

[52] Hub J.S., De Groot B.L., Van Der Spoel D. g_wham A free weighted histogram analysis implementation including robust error and autocorrelation estimates. J. Chem. Theory Comput. 2010; 6:3713–3720.

[53] Zwanzig R.W. High‐temperature equation of state by a perturbation method. I. nonpolar gases. J. Chem. Phys. 1954; 22:1420–1426.

[54] Bennett C.H. Efficient estimation of free energy differences from Monte Carlo data. J. Comput. Phys. 1976; 22:245–268.

[55] Hess B., Kutzner C., Van Der Spoel D., Lindahl E. GROMACS 4: algorithms for highly efficient, load-balanced, and scalable molecular simulation. J. Chem. Theory Comput. 2008; 4:435–447. 26620784 10.1021/ct700301q

[56] Liu P., Dehez F.O., Cai W., Chipot C. A toolkit for the analysis of free-energy perturbation calculations. J. Chem. Theory Comput. 2012; 8:2606–2616. 26592106 10.1021/ct300242f

[57] Hess B., Bekker H., Berendsen H.J., Fraaije J.G. LINCS: a linear constraint solver for molecular simulations. J. Comput. Chem. 1997; 18:1463–1472.

[58] Thomen P., Lopez P.J., Heslot F. Unravelling the mechanism of RNA-Polymerase forward motion by using mechanical force. Phys. Rev. Lett. 2005; 94:128102. 15903965 10.1103/PhysRevLett.94.128102

[59] Sosunov V., Sosunova E., Mustaev A., Bass I., Nikiforov V., Goldfarb A. Unified two-metal mechanism of RNA synthesis and degradation by RNA polymerase. EMBO J. 2003; 22:2234–2244. 12727889 10.1093/emboj/cdg193 PMC156065

[60] Steitz T.A., Steitz J.A. A general two-metal-ion mechanism for catalytic RNA. Proc. Natl. Acad. Sci. U.S.A. 1993; 90:6498–6502. 8341661 10.1073/pnas.90.14.6498 PMC46959

[61] Wu S., Wang J., Pu X., Li L., Li Q. T7 RNA polymerase discriminates correct and incorrect nucleoside triphosphates by free energy. Biophys. J. 2018; 114:1755–1761. 29694856 10.1016/j.bpj.2018.02.033 PMC5937113

[62] MacKerell A.D., Bashford D., Bellott M., Dunbrack R.L., Evanseck J.D., Field M.J., Fischer S., Gao J., Guo H., Ha S. et al. All-Atom empirical potential for molecular modeling and dynamics studies of proteins. J. Phys. Chem. B. 1998; 102:3586–3616. 24889800 10.1021/jp973084f

[63] Mackerell A.D. Jr, Feig M., Brooks C.L. III Extending the treatment of backbone energetics in protein force fields: limitations of gas‐phase quantum mechanics in reproducing protein conformational distributions in molecular dynamics simulations. J. Comput. Chem. 2004; 25:1400–1415. 15185334 10.1002/jcc.20065

[64] Zoete V., Cuendet M.A., Grosdidier A., Michielin O. SwissParam: a fast force field generation tool for small organic molecules. J. Comput. Chem. 2011; 32:2359–2368. 21541964 10.1002/jcc.21816

[65] Vanommeslaeghe K., Hatcher E., Acharya C., Kundu S., Zhong S., Shim J., Darian E., Guvench O., Lopes P., Vorobyov I. CHARMM general force field: A force field for drug‐like molecules compatible with the CHARMM all‐atom additive biological force fields. J. Comput. Chem. 2010; 31:671–690. 19575467 10.1002/jcc.21367 PMC2888302

[66] Yu W., He X., Vanommeslaeghe K., MacKerell A.D. Jr Extension of the CHARMM general force field to sulfonyl‐containing compounds and its utility in biomolecular simulations. J. Comput. Chem. 2012; 33:2451–2468. 22821581 10.1002/jcc.23067 PMC3477297

[67] Dupradeau F.-Y., Pigache A., Zaffran T., Savineau C., Lelong R., Grivel N., Lelong D., Rosanski W., Cieplak P. The REd. Tools: Advances in RESP and ESP charge derivation and force field library building. Phys. Chem. Chem. Phys. 2010; 12:7821–7839. 20574571 10.1039/c0cp00111b PMC2918240

[68] Yu J., Oster G. A small Post-translocation energy bias aids nucleotide selection in T7 RNA polymerase transcription. Biophys. J. 2012; 102:532–541. 22325276 10.1016/j.bpj.2011.12.028 PMC3274829

[69] Da L.-T., E C., Shai Y., Wu S., Su X.-D., Yu J. T7 RNA polymerase translocation is facilitated by a helix opening on the fingers domain that may also prevent backtracking. Nucleic Acids Res. 2017; 45:7909–7921. 28575393 10.1093/nar/gkx495 PMC5737862

