# Supplementary material for: Determining selection free energetics from nucleotide pre-insertion to insertion in viral T7 RNA polymerase transcription fidelity control
Source: Nucleic Acids Res. 2019 Mar 27;47(9):4721–35. doi: 10.1093/nar/gkz213 (PMC6511863; doi:10.1093/nar/gkz213)
Supplement: Supplementary Data [file gkz213_supplemental_files.zip › T7RNAP-NTPselection-SI-second-revision-submit.pdf]

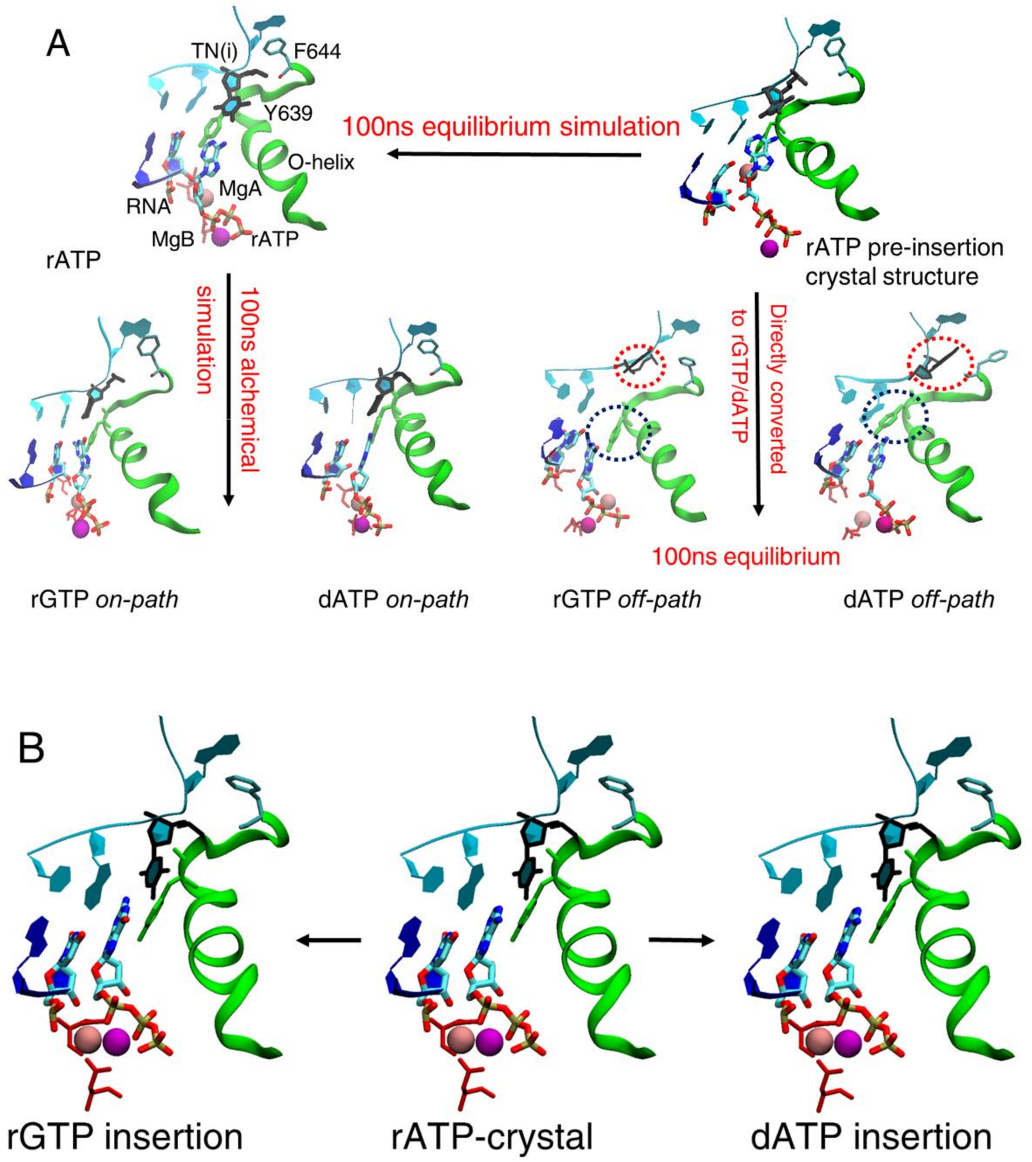

Figure S1. The illustration of procedures on preparing the initial pre-insertion and final insertion structures for the umbrella sampling simulations. (A) The cognate rATP pre-insertion structure was obtained after an equilibration MD simulation of 100 ns (top), starting from the crystal structure of the T7 RNAP pre-insertion complex (PDB 1S0V) (1). The procedures for making the dATP and rGTP *on-path* structures are shown (bottom left): After the 100-ns equilibrium MD simulation of the rATP pre-insertion complex, alchemical simulations were conducted to transform rATP gradually into dATP and rGTP (2), respectively. The procedures for making the dATP and rGTP *off-path* structures are shown as well (bottom right): The cognate rATP in the crystal structure was converted into the non-cognate rGTP and dATP, respectively, and after that, 100-ns equilibrium simulations were conducted for the respective non-cognate pre-insertion complexes (3). (B) For the final insertion structures, the cognate rATP structure was obtained from the crystal substrate complex (PDB: 1S76) (4). The non-cognate rGTP and dATP insertion structures were constructed by converting rATP in the crystal structure into dATP and rGTP along with equilibrium simulations, respectively.

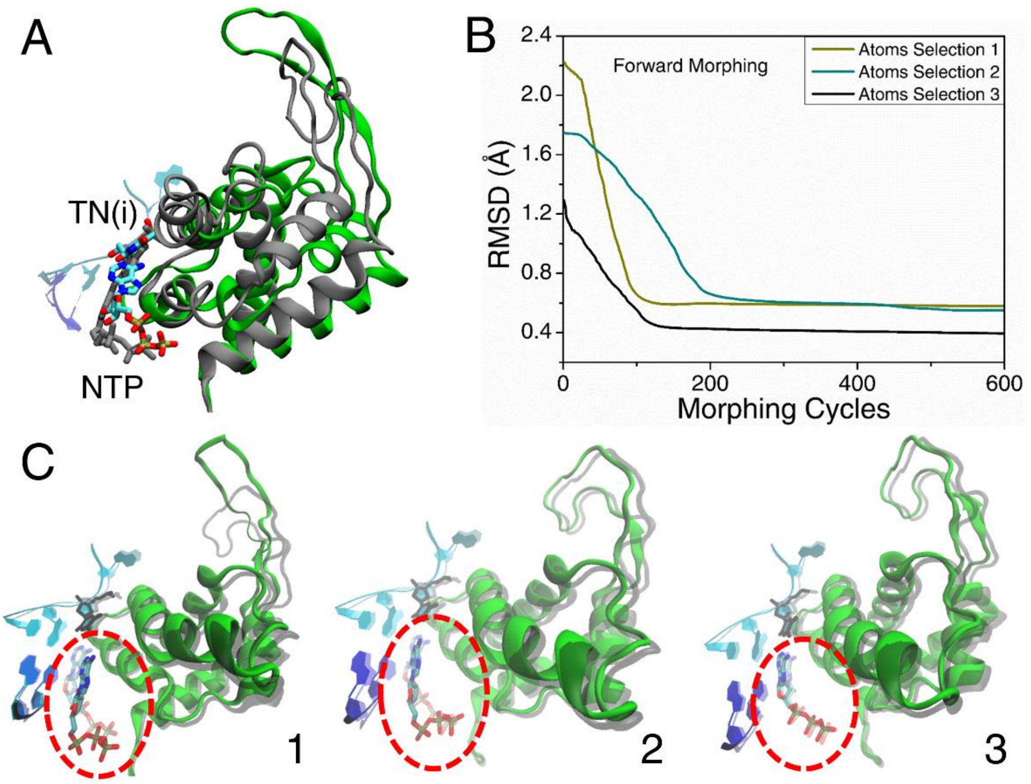

Figure S2. The morphed region for launching the nucleotide insertion pathway along the collective reaction coordinate $\delta rmsd$. (A) The Cα atoms of five helices on the fingers subdomain (residue 627 to 639, 568 to 589, 612 to 624, 642 to 660, and 669 to 687) and heavy atoms of substrate rATP/rGTP/dATP and template TN(i) are selected. The substrate insertion structure (PDB: 1S76) (4) is shown as a final reference state (colored in gray). The O-helix on the fingers subdomain of the pre-insertion crystal structure (PDB:1S0V; colored in green) is in the open configuration (measured ~15°), while in the insertion crystal structure, it is in the closed configuration (measured ~5°). Besides, the base of the incoming nucleotide doesn't pair well with the template TN(i) yet in the pre-insertion crystal structure, the phosphate group of NTP doesn't insert into the active site as well. While in the insertion structure, the binding nucleotide inserts well into the active site and makes nice WC base-pairing with the template TN(i). (B) The morphing results for the nucleotide rATP insertion by including different sets of atoms. The RMSD measured in reference to the target insertion structure along the forward insertion pathway, generated by using a modified Climber algorithm (5). The atom selection 1 and 2 refer to the ones with additional atoms from DNA/RNA strands (see text), while the atom selection 3 refers to our currently used one. The RMSD values of the selection 1 and 2 cannot reach as low as that of the selection 3 (~ 0.4 Å) toward the end of the morphing simulation. (C) The alignments between the final structures reached by individual morphing simulations for the selections 1 to 3 (opaque in green, from left to right) and the target insertion structure (transparent in gray). The inserted NTP is circled for an easy inspection. Only for the selection 3, the final insertion structure is well reached.

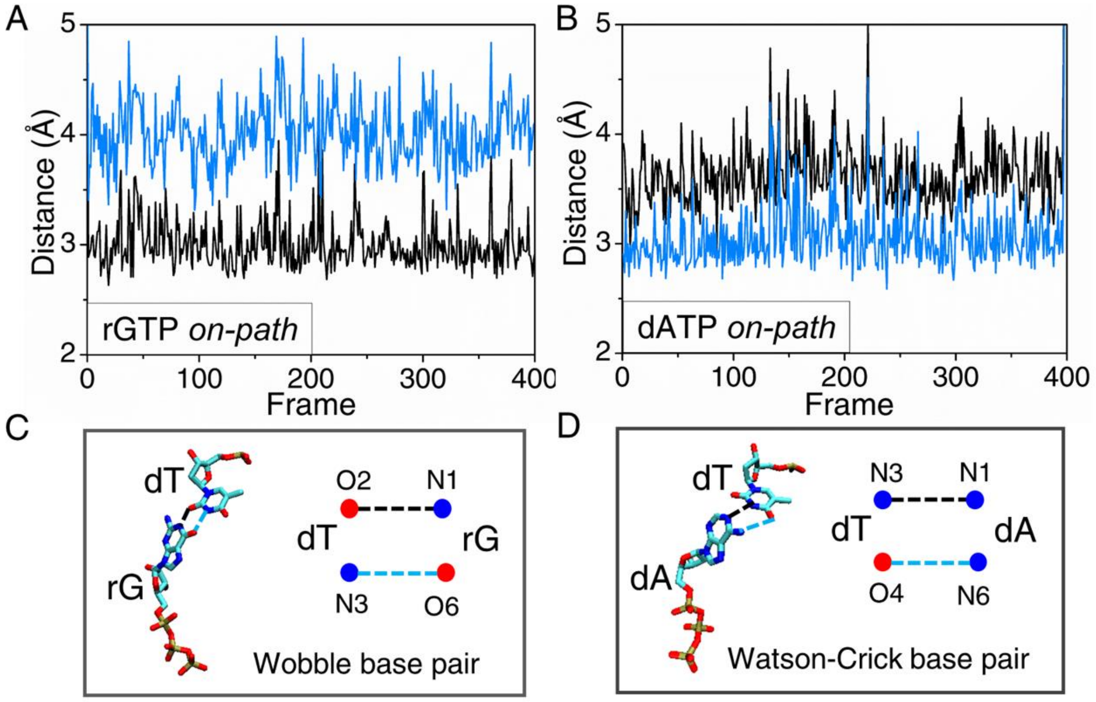

Figure S3. Base pairings between the *on-path* non-cognate nucleotide and the template TN(i). The hydrogen-bond distances between rGTP (A) /dATP (B) and template TN(i) obtained from the 40-ns umbrella sampling simulation of the pre-insertion complex (see config 1 from main Figure 3 and Figure 6). (C) The wobble base pairing convention between the template TN(i) (dTTP or dT) and the substrate rGTP (rG). (D) The Watson-Crick base pairing convention between the template dT and the substrate dATP (dA).

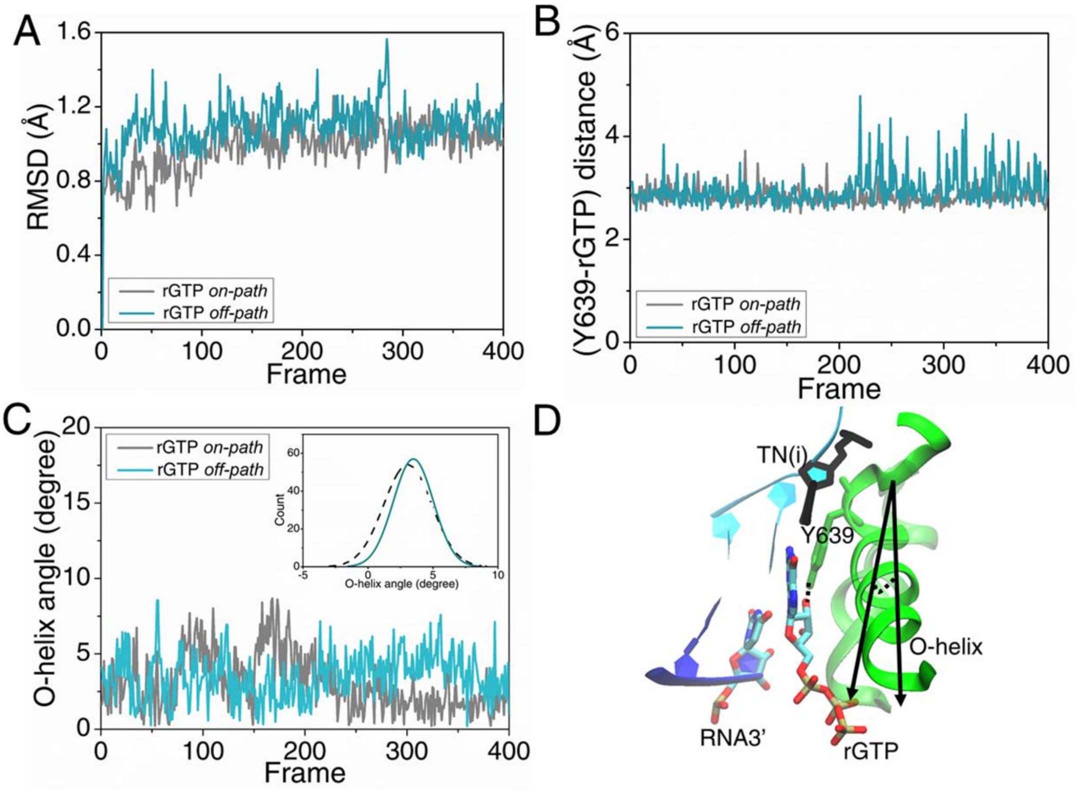

Figure S4. Structural measures showing similarities between the final rGTP *on-path* and *off-path* insertion configurations (config 5 from main Figure 3 and Figure 4). (A) The root-mean-square deviations (RMSDs) for the heavy atoms of the fingers subdomain with respect to the substrate structure. (B) The respective distances between Tyr639-OH and rGTP-2'OH in the *on-path* and *off-path* insertion configurations. (C) The respective O-helix rotation angles in the rGTP *on-path* and *off-path* insertion configurations. In T7 RNAP, residues 623 to 642 are assigned to the O-helix, and the vector-line defining the orientation of the O-helix is obtained by connecting the centers of mass (COMs) of residue 623 to 633 and residue 633 to 642. Then the O-helix angle was measured between the current vector lines and the one in the substrate-insertion state as reference. (D) The structural illustrations of the above distance and the O-helix rotation angle.

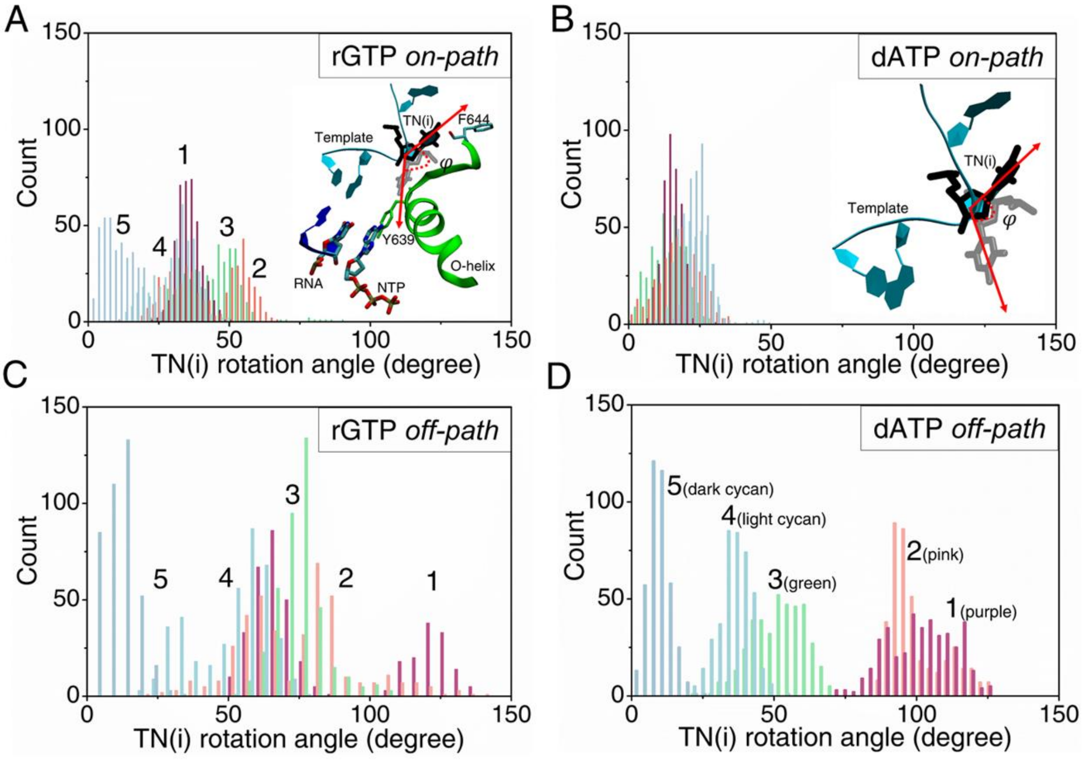

Figure S5. The histograms of the template nt TN(i) base rotation angles obtained from the umbrella sampling simulations of the *on-path* (A & B) and *off-path* (C & D) insertion of the non-cognate rGTP and dATP. The base rotation angle φ is illustrated in the structural views in the inset of (A) and (B). A vector line defining the orientation of the base is obtained by connecting the center of mass of the sugar and base of TN(i); the angle φ was measured between the current vector line (base shown in dark) and the one in the substrate-insertion state as the reference (the base shown in gray). Note that the histograms are provided for simulation windows 1 to 5 in the PMF curve (see main).

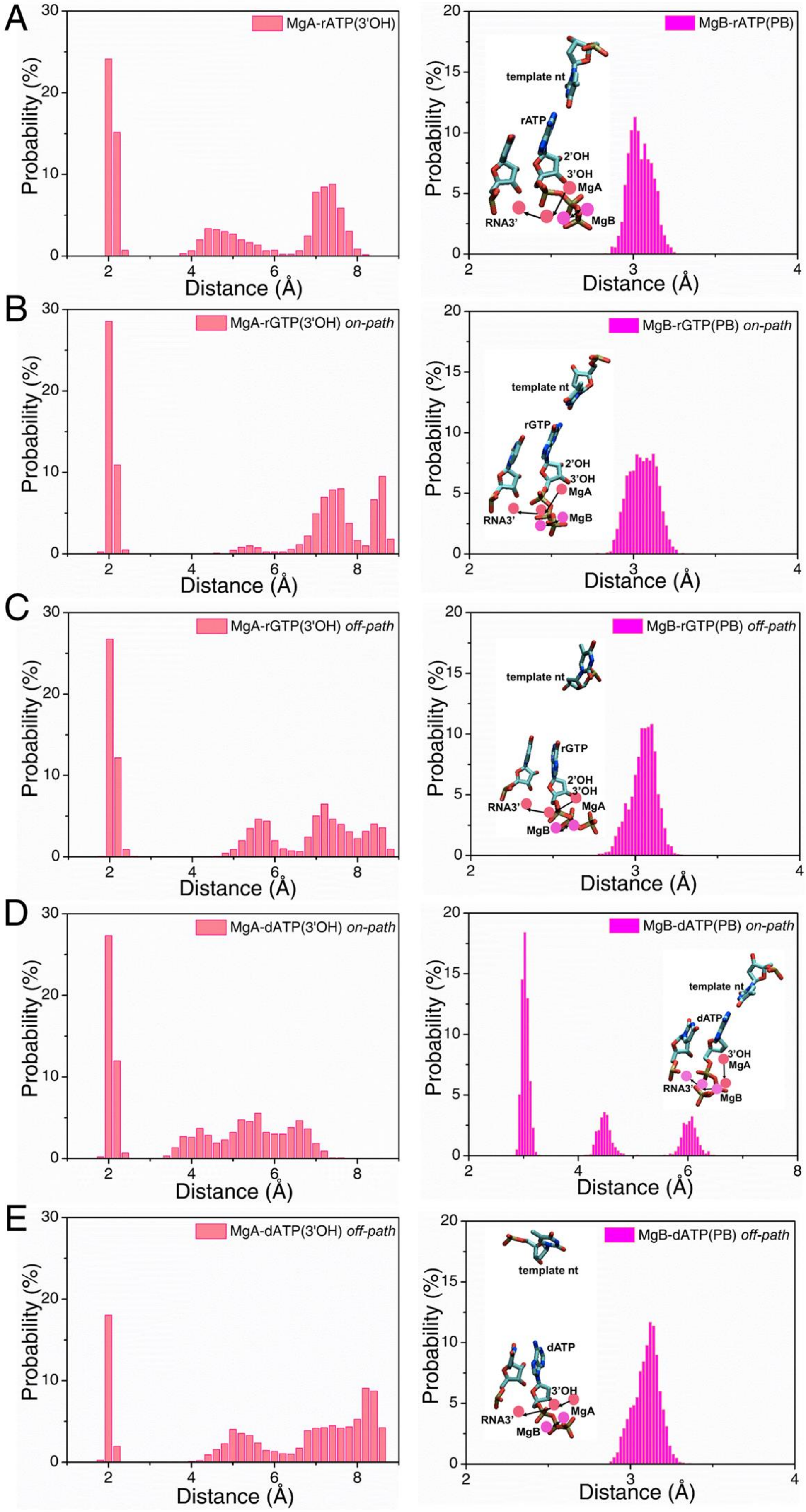
A
MgA-rATP(3'OH)
MgB-rATP(PB)
template nt
rATP
2'OH
3'OH
MgA
MgB
RNA3'
B
MgA-rGTP(3'OH) on-path
MgB-rGTP(PB) on-path
template nt
rGTP
2'OH
3'OH
MgA
MgB
RNA3'
C
MgA-rGTP(3'OH) off-path
MgB-rGTP(PB) off-path
template nt
rGTP
2'OH
3'OH
MgA
MgB
RNA3'
D
MgA-dATP(3'OH) on-path
MgB-dATP(PB) on-path
template nt
dATP
3'OH
MgA
MgB
RNA3'
E
MgA-dATP(3'OH) off-path
MgB-dATP(PB) off-path
template nt
dATP
3'OH
MgA
MgB
RNA3'
Probability (%)
Distance (Å)

Figure S6. Transitions of MgA and MgB captured via umbrella sampling simulations for various nucleotide substrates from pre-insertion to insertion. The probability distribution of distances, measured between MgA and 3'OH of the substrate NTPs, are used to describe the transitions of MgA (in pink). For MgB, the distance was measured between MgB and the β phosphate or PB atom of NTP (in magenta). For the cognate rATP (A) and the non-cognate rGTP *on-path* (B) & *off-path* (C), MgA moves from an initial (near the pre-insertion rNTP 3'OH) to the final (between the RNA3'-end and rNTP substrates) via an intermediate position (close to the α phosphate or PA atom), while MgB fluctuates around the β phosphate or PB atom. (D) For the non-cognate dATP *on-path*, both MgA and MgB move via some transition from the pre-insertion configuration to the insertion one, while MgA ends up close to β and γ phosphate (or PB and PC atoms), and MgB associates closely to the RNA 3'-end in the end, switching from the presumable positioning. (E) For the non-cognate dATP *off-path*, MgA stays far from dATP-3'OH at the beginning of the pre-insertion equilibrium condition, and then moves via only one intermediate state (close to dATP-3'OH atom), while the final position is still far from the RNA 3'-end. MgB keeps similar positioning as in (A-C).

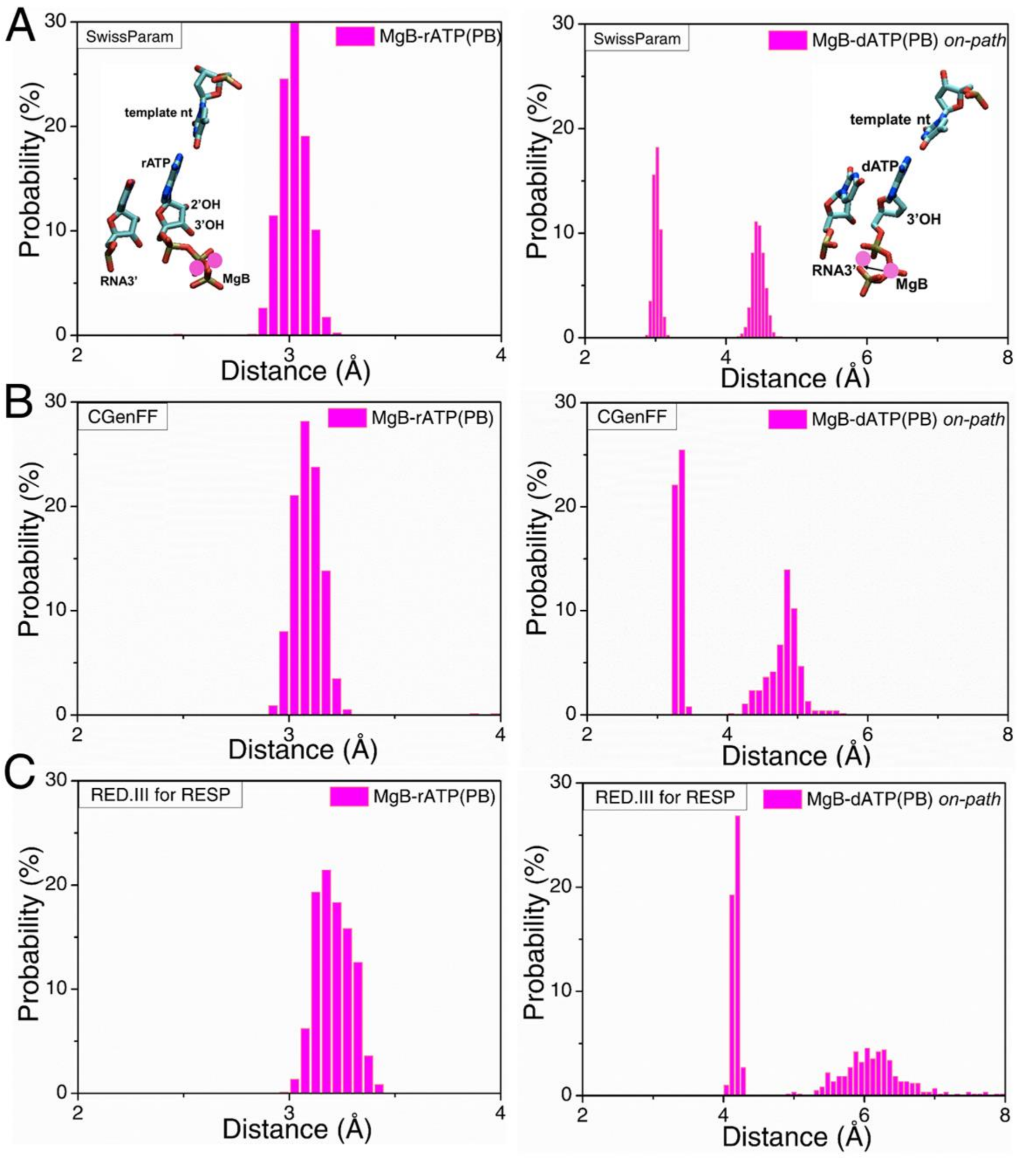

Figure S7. Transitions of MgB captured via umbrella sampling simulations for nucleotides rATP and dATP in config 3 and config 4. (A) Transitions of MgB in rATP (left) and dATP (right), respectively, by using SwissParam (6) to generate the CHARMM force field. (B) Transitions of MgB in rATP (left) and dATP (right), respectively, by using CGenFF (7,8) to generate the CHARMM force field. (C) Transitions of MgB in rATP (left) and dATP (right), respectively, by using RED.III software (9) to calculate the (rATP & MgB) and (dATP & MgB) group RESP charges, and using antechamber to obtain the corresponding groups' AMBER force field (10). All the variations on the force field implementations can well reproduce the phenomenon that MgA and MgB switch positioning during the dATP *on-path* insertion process.

A

RESP charge for dATP and MGB, respectively			
ATOM	RESP	ATOM	RESP
MG	2.0000	C2'	-0.0854
O1G	-0.9526	H20	0.0718
PG	1.2650	H21	0.0718
O2G	-0.9526	C1'	0.0431
O3G	-0.9526	H10	0.1838
O3B	-0.5322	N9	-0.0268
PB	1.3852	C8	0.1607
O1B	-0.8894	H8	0.1877
O2B	-0.8894	N7	-0.6175
O3A	-0.5689	C5	0.0725
PA	1.2564	C4	0.3800
O1A	-0.8799	N3	-0.7417
O2A	-0.8799	C2	0.5716
O5'	-0.5987	H2	0.0598
C5'	-0.0069	N1	-0.7624
H50	0.0754	C6	0.6897
H51	0.0754	N6	-0.9123
C4'	0.1629	H60	0.4167
H40	0.1176	H61	0.4167
O4'	-0.3691	O3'	-0.6549
C3'	0.0713	H3'	0.4396
H30	0.0985		

B

RESP charge for (dATP & MGB) group			
ATOM	RESP	ATOM	RESP
MG	1.6457	C2'	-0.0681
O1G	-0.2673	H20	0.0527
PG	1.5713	H21	0.0527
O2G	-0.2594	C1'	0.0752
O3G	-0.8701	H10	0.1605
O3B	-0.5953	N9	-0.0524
PB	1.4415	C8	0.1220
O1B	-0.6470	H8	0.2029
O2B	-0.9171	N7	-0.5753
O3A	-0.6624	C5	0.0325
PA	1.4185	C4	0.4619
O1A	-0.9428	N3	-0.7717
O2A	-0.6869	C2	0.5766
O5'	-0.4815	H2	0.0618
C5'	0.0180	N1	-0.7568
H50	0.1029	C6	0.7049
H51	0.1029	N6	-0.9193
C4'	0.0397	H60	0.4117
H40	0.1469	H61	0.4117
O4'	-0.3968	O3'	-0.6777
C3'	0.1503	H3'	0.4385
H30	0.1446		

Table S1. (A) The RESP charges for dATP and MgB, respectively, obtained directly from the AMBER force field (10). (B) The RESP charges for (dATP & MgB) as a group. We used RED.III software (9) to calculate the (dATP & MgB) group RESP charges.

A

RESP charge for rATP and MGB, respectively			
ATOM	RESP	ATOM	RESP
MG	2.0000	C2'	0.0670
O1G	-0.9526	H20	0.0972
PG	1.2650	C1'	0.0394
O2G	-0.9526	H10	0.2007
O3G	-0.9526	N9	-0.0251
O3B	-0.5322	C8	0.2006
PB	1.3852	H8	0.1553
O1B	-0.8894	N7	-0.6073
O2B	-0.8894	C5	0.0515
O3A	-0.5689	C4	0.3053
PA	1.2564	N3	-0.6997
O1A	-0.8799	C2	0.5875
O2A	-0.8799	H2	0.0473
O5'	-0.5987	N1	-0.7615
C5'	0.0558	C6	0.7009
H50	0.0679	N6	-0.9019
H51	0.0679	H60	0.4115
C4'	0.1065	H61	0.4115
H40	0.1174	O3'	-0.6541
O4'	-0.3548	H3'	0.4376
C3'	0.2022	O2'	-0.6139
H30	0.0615	H2'	0.4186

B

RESP charge for (rATP & MGB) group			
ATOM	RESP	ATOM	RESP
MG	1.6556	C2'	0.1430
O1G	-0.2725	H20	0.0670
PG	1.3083	C1'	0.0750
O2G	-0.2945	H10	0.1661
O3G	-0.8924	N9	-0.0301
O3B	-0.5874	C8	0.1784
PB	1.4468	H8	0.1559
O1B	-0.6617	N7	-0.5727
O2B	-0.9241	C5	0.0925
O3A	-0.5233	C4	0.2375
PA	1.0094	N3	-0.4803
O1A	-0.8025	C2	0.3860
O2A	-0.4929	H2	0.0941
O5'	-0.2654	N1	-0.6652
C5'	-0.0092	C6	0.6375
H50	0.1171	N6	-0.8650
H51	0.1171	H60	0.4026
C4'	0.1615	H61	0.4026
H40	0.0935	O3'	-0.5922
O4'	-0.4142	H3'	0.4146
C3'	0.0836	O2'	-0.5439
H30	0.1159	H2'	0.3279

Table S2. (A) The RESP charges for rATP and MgB, respectively, obtained directly from the AMBER force field (10). (B) The RESP charges for (rATP & MgB) as a group. We used RED.III software (9) to calculate the (rATP & MgB) group RESP charges.

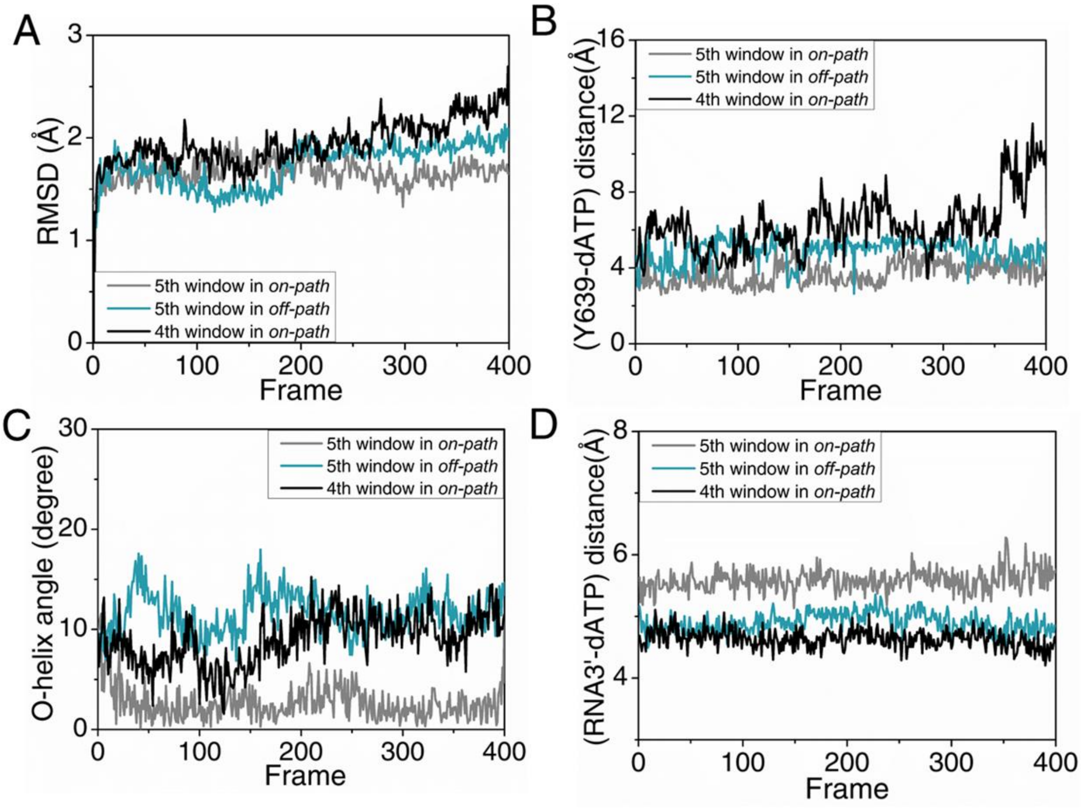

Figure S8. Structural measures of the dATP *off-path* insertion configuration and the final two *on-path* configurations. (A) The RMSDs for the heavy atoms of the fingers subdomain for the dATP *off-path* config 5 and the two final *on-path* config 4 and 5 (see main Figure 6 and Figure 7) with respect to the substrate reference structure. (B) The respective distances between Tyr639-OH and dATP-3'OH. (C) The respective O-helix rotation angles. (D) The respective distances between RNA 3'-end (P atom) and dATP (PA atom).

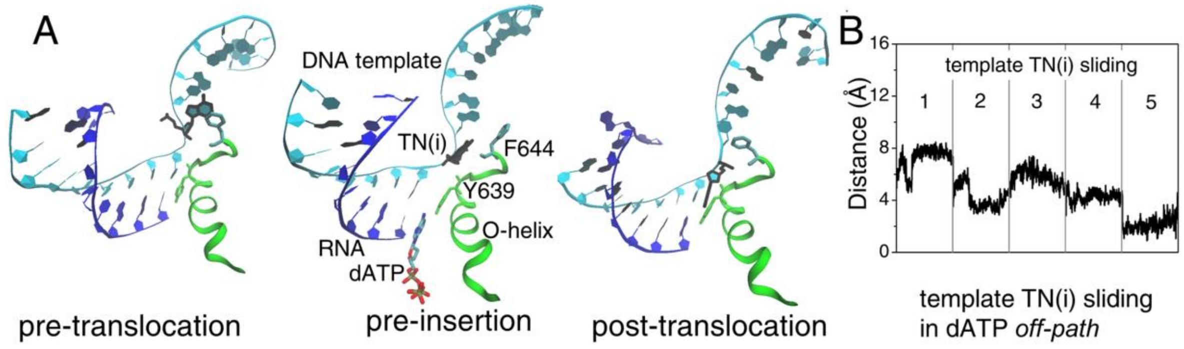

Figure S9. Comparing the dATP *off-path* pre-insertion structure (around the active site) with that of the pre-translocation and post-translocation structures. (A) In the obtained dATP *off-path* pre-insertion complex (*middle*), the template TN(i) has been 'pushed' backward to an translocation intermediate position right in between the pre-translocation (*left*) and the post-translocation *(right*) structures (PDB codes: 1MSW and 1S77) (4,11). (B) The distance of template TN(i) sliding from the dATP *off-path* pre-insertion state to substrate-insertion state during the umbrella sampling simulation. The distance is measured between the current template TN(i) and the one in the substrate insertion reference structure, from config 1 to 5 (see main Figure 7).

Summary on stepwise selection free energetics from nucleotide pre-insertion to insertion

Upon previous simulation studies, the NTP dissociation free energy barriers differences (Δ_b^-) between the cognate and the *off-path* non-cognate species at the pre-insertion site were ~4 k_BT (12); Δ_b^- is assumed to be zero between the cognate and the *on-path* non-cognate dissociation. According to our umbrella sampling simulation results, the free energy barrier for the rATP insertion is ΔE_{in}^c~3 k_BT, and those for the rGTP/dATP *on-path* and rGTP/dATP *off-path* insertion are ΔE_{in}^{nc} ~ 4 k_BT / 6 k_BT, 7 k_BT / 4 k_BT, respectively; then the corresponding insertion selection energies Δ_{in}^+ (or $\Delta E_{in}^{nc} - \Delta E_{in}^c$) are 1 k_BT / 3 k_BT for the rGTP/dATP *on-path,* and 4 k_BT / 1 k_BT for the rGTP/dATP *off-path*. Similarly, from the PMFs, we obtained the free energy barrier for reversal of the insertion of the cognate rATP as ΔE_{rev}^c ~ 6 k_BT, while those for the inserted non-cognate rGTP/dATP *on-path* and the rGTP/dATP *off-path* rejection were calculated respectively as ΔE_{rev}^{nc}~ 8 k_BT / 7 k_BT and 10 k_BT / 5 k_BT. Correspondingly, the selection energies via the reversal of the insertion Δ_{in}^- (or $\Delta E_{rev}^c - \Delta E_{rev}^{nc}$) were -2 k_BT / -1 k_BT for the rGTP/dATP *on-path,* and -4 k_BT / 1 k_BT for the rGTP/dATP *off-path*. Furthermore, as we cannot simulate the catalytic step in our classical MD simulations, the free energy barrier differences Δ_c^+ could only be inferred by using the CME approach. To fit with an elongation error rate for rGTP at *Err*~10^{-4} (13) and for dATP at *Err*~10^{-2} (14), as experimentally detected, one then infers that Δ_c^+~ 7 k_BT for the base mismatch rGTP and Δ_c^+~ 0 k_BT for the sugar deficiency dATP.

In order to estimate the weights for the *on-path* and *off-path* insertion processes of the non-cognate nucleotides, one writes

$$\frac{p_{on}}{p_{off}} = e^{-(\Delta E_a^{on} - \Delta E_a^{off})} \quad \text{(S1)}$$

$$p_{on} + p_{off} = 1 \quad \text{(S2)}$$

where p_{on} and p_{off} denote the weights/percentiles for the *on-path* and *off-path,* respectively, while ΔE_a^{on} and ΔE_a^{off} denote the corresponding association free energy barriers. For rGTP, as shown in main Figure 5, ΔE_a^{on} is ~ 5.3 k_BT, while ΔE_a^{off} is zero, or say, no association barrier, so that $p_{on} =$ 0.5%, $p_{off} = 99.5\%$. For dATP, as shown in main Figure 8, ΔE_a^{on} ~ ΔE_a^{off} ~ 1.2 k_BT so that p_{on}~50% and p_{off}~50%. The obtained selection free energetics and the selection strengths were summarized in Table S3**.**

	Error rate conduction for non-cognate substrate species (rGTP/dATP)										
	Δ_b^- (k_BT)	η_{III}^-	Δ_{in}^+ (k_BT)	η_{III}^+	Δ_{in}^- (k_BT)	η_{IV}^-	Δ_c^+ (k_BT)	η_{IV}^+	Error rate	Approx weight of the path	Error rate *Err*
rGTP *on-path*	0	1	1	3	-2	0.13	7	1000	8*10^{-2}	0.5%	4*10^{-4}
rGTP *off-path*	4	60	4	60	-4	0.02	7	1000	4*10^{-4}	99.5%	3*10^{-4}

dATP *on-path*	0	1	3	20	-1	0.3	0	1	$1.6*10^{-1}$	50%	$8*10^{-2}$
dATP *off-path*	4	60	1	3	1	3	0	1	$2.5*10^{-2}$	50%	$1*10^{-2}$

Table S3. Stepwise selection free energetics ($\Delta^{\pm}$) and selection strength ($\eta^{\pm}$) obtained from the MD simulation and the CME approach in the five-state NAC of T7 RNAP elongation.

Movie S1

The cognate rATP insertion dynamics from configuration or config 1 to 5 (see main Figure 2 and caption)

Movie S2

The non-cognate rGTP *on-path* insertion dynamics from config 1 to 5 (see main Figure 3 and caption)

Movie S3

The non-cognate rGTP *off-path* insertion dynamics from config 1 to 5 (see main Figure 4 and caption)

Movie S4

The non-cognate dATP *on-path* insertion dynamics from config 1 to 5 (see main Figure 6 and caption)

Movie S5

The non-cognate dATP *off-path* insertion dynamics from config 1 to 5 (see main Figure 7 and caption)

REFERENCES

1. Temiakov, D., Patlan, V., Anikin, M., McAllister, W.T., Yokoyama, S. and Vassylyev, D.G. (2004) Structural Basis for Substrate Selection by T7 RNA Polymerase. *Cell*, **116**, 381-391.
2. E, C., Duan, B. and Yu, J. (2017) Nucleotide Selectivity at a Preinsertion Checkpoint of T7 RNA Polymerase Transcription Elongation. *J Phys Chem B*, **121**, 3777-3786.
3. Duan, B., Wu, S., Da, L.-T. and Yu, J. (2014) A critical residue selectively recruits nucleotides for T7 RNA polymerase transcription fidelity control. *Biophysical journal*, **107**, 2130-2140.
4. Yin, Y.W. and Steitz, T.A. (2004) The structural mechanism of translocation and helicase activity in T7 RNA polymerase. *Cell*, **116**, 393-404.
5. Weiss, D.R. and Levitt, M. (2009) Can morphing methods predict intermediate structures? *Journal of molecular biology*, **385**, 665-674.
6. Zoete, V., Cuendet, M.A., Grosdidier, A. and Michielin, O. (2011) SwissParam: a fast force field generation tool for small organic molecules. *Journal of computational chemistry*, **32**, 2359-2368.
7. Vanommeslaeghe, K., Hatcher, E., Acharya, C., Kundu, S., Zhong, S., Shim, J., Darian, E., Guvench, O., Lopes, P. and Vorobyov, I. (2010) CHARMM general force field: A force field for drug‐like molecules compatible with the CHARMM all‐atom additive biological force fields. *Journal of computational chemistry*, **31**, 671-690.
8. Yu, W., He, X., Vanommeslaeghe, K. and MacKerell Jr, A.D. (2012) Extension of the CHARMM general force field to sulfonyl‐containing compounds and its utility in biomolecular simulations. *Journal of computational chemistry*, **33**, 2451-2468.
9. Dupradeau, F.-Y., Pigache, A., Zaffran, T., Savineau, C., Lelong, R., Grivel, N., Lelong, D., Rosanski, W. and Cieplak, P. (2010) The REd. Tools: Advances in RESP and ESP charge derivation and force field library building. *Physical Chemistry Chemical Physics*, **12**, 7821-7839.
10. Hornak, V., Abel, R., Okur, A., Strockbine, B., Roitberg, A. and Simmerling, C. (2006) Comparison of multiple Amber force fields and development of improved protein backbone parameters. *Proteins: Structure, Function, and Bioinformatics*, **65**, 712-725.
11. Yin, Y.W. and Steitz, T.A. (2002) Structural basis for the transition from initiation to elongation transcription in T7 RNA polymerase. *Science*, **298**, 1387-1395.
12. Wu, S., Wang, J., Pu, X., Li, L. and Li, Q. (2018) T7 RNA Polymerase Discriminates Correct and Incorrect Nucleoside Triphosphates by Free Energy. *Biophysical Journal*, **114**, 1755-1761.
13. Sultana, S., Solotchi, M., Ramachandran, A. and Patel, S.S. (2017) Transcriptional fidelities of human mitochondrial POLRMT, yeast mitochondrial Rpo41, and phage T7 single-subunit RNA polymerases. *J Biol Chem*, **292**, 18145-18160.
14. Huang, J., Brieba, L.G. and Sousa, R. (2000) Misincorporation by Wild-Type and Mutant T7 RNA Polymerases: Identification of Interactions That Reduce Misincorporation Rates by Stabilizing the Catalytically Incompetent Open Conformation. *Biochemistry*, **39**, 11571-11580.
